# Dynamic Energy Budget model for *E. coli* growth in carbon and nitrogen limitation conditions

**DOI:** 10.1007/s00253-024-13245-9

**Published:** 2024-07-05

**Authors:** Konrad Matyja, Magdalena Lech

**Affiliations:** Faculty of Chemistry, Department of Micro, Nano, and Bioprocess Engineering, Wroclaw University of Science and Technology, Wybrzeże Wyspiańskiego 27, 50-370 Wrocław, Poland

**Keywords:** Elemental composition, Mechanistic models, Bacteria, Growth phases, Batch culture

## Abstract

**Abstract:**

The simulations and predictions obtained from mathematical models of bioprocesses conducted by microorganisms are not overvalued. Mechanistic models are bringing a better process understanding and the possibility of simulating unmeasurable variables. The Dynamic Energy Budget (DEB) model is an energy balance that can be formulated for any living organism and can be classified as a structured model. In this study, the DEB model was used to describe *E. coli* growth in a batch reactor in carbon and nitrogen substrate limitation conditions. The DEB model provides a possibility to follow the changes in the microbes’ cells including their elemental composition and content of some important cell ingredients in different growth phases in substrate limitation conditions which makes it more informative compared to Monod’s model. The model can be used as an optimal choice between Monod-like models and flux-based approaches.

**Key points:**

*• The DEB model can be used to catch changes in elemental composition of E. coli*

*• Bacteria batch culture growth phases can be explained by the DEB model*

*• The DEB model is more informative compared to Monod’s based models*

**Supplementary Information:**

The online version contains supplementary material available at 10.1007/s00253-024-13245-9.

## Introduction

The number of industrial bioprocesses conducted by host microorganisms seems to be growing (Nielsen et al. 2022). Each of these processes, regardless if it is a continuous, semi-continuous, or batch process, needs to be designed, controlled, and optimized. The simulations and predictions obtained from mathematical models of these processes are not overvalued in these aspects (Luo et al. 2021; Rathore et al. 2021).

There are two main approaches to bioprocess modeling (Luo et al. 2021). First, it is quite popular nowadays based on data-driven models including artificial intelligence, and second based on mechanistic models. Data-driven models as the name suggests need a lot of data to be calibrated and used to simulate and optimize different processes (Luo et al. 2021). This is one important disadvantage of this approach as some of the process variables are difficult or even impossible to be measured. Furthermore, they do not explain the mechanism of the process, unlike mechanistic models. Mechanistic models are derived from known physical, chemical, and biological laws and/or phenomenological relationships and are bringing a better process understanding and the possibility of simulating unmeasurable variables (Luo et al. 2021). In this group of models, we can distinguish between flux-based and kinetic-based models.

Flux-based models can be used to describe the biochemical pathways in living organisms. A good example is a method called flux balance analysis (FBA) (Orth et al. 2010). It was shown that it can be used to modify and optimize culture medium composition or even bacterial strain phenotype by genetic modifications in order to obtain high bioprocess efficiency (Lee et al. 2006; Raman and Chandra 2009; Swayambhu et al. 2020). These complex models can provide a huge amount of information about microorganisms’ physiology; however, in most cases, they are based on steady-state assumption, and they cannot be used to model growth kinetics (Orth et al. 2010; Stryjewski et al. 2015). On the other hand, there are kinetic-based models which are often based on Monod’s equation with different modifications (Monod 1949). This type of modeling often found a lot of applications including organic substances, ammonia nitrogen, and phosphorous removal by activated sludge (Hauduc et al. 2013; Henze et al. 2000; Hu et al. 2007).

In these kinds of models, unlike the flux-based models, the biomass of bacteria is treated as a single component, and the whole complexity of living organisms is brought to one variable. This simplification is reasonable in many cases; however, in many others, more sophisticated models have to be applied to catch observed phenomena (Jager et al. 2006; Stryjewski et al. 2015). On the other hand, too complex modes can have a large number of variables and parameters that can be difficult to measure or estimate (Luo et al. 2021). Therefore, the model complexity has to be chosen according to specific needs arising from certain bioprocess features.

The basic idea behind increasing the complexity of the model is to divide microorganisms’ cells into compartments and describe them with different variables. These kinds of models are often called structured models (Morchain & Fonade, 2009; Stryjewski et al. 2015). One of the first two-compartment models used to explain such phenomena as changes in size and composition of cells at higher growth rates, and cell division after nutrients were removed, and many others, was proposed by Williams (Williams 1967).

It seems that the application of structured models in the case of processes with substrate limitations is a reasonable idea. Multiple substrate limitations can influence the kinetics and stoichiometry of bacteria growth and therefore it can have a large impact on the efficiency of bioprocess (Zinn et al. 2004). Depending on the limiting substrate concentrations and substitutability, different effects on the growth rate can be observed (Zinn et al. 2004). Moreover, the elemental composition of microorganisms, DNA, RNA, enzymes, and other intracellular components contents can be changed (Pramanik and Keasling 1997; Zinn et al. 2004).

Therefore, in this study, we evaluate the Dynamic Energy Budget (DEB) model used to describe *E. coli* growth in a carbon and nitrogen substrate limitation. DEB theory and model is a complex mass and energy balance prepared for living organisms (Kooijman 2010). It describes how an organism obtains mass and energy from the environment and uses it to maintain life, grow, and reproduce. It can be classified as a structured model because it assumes that organisms can be divided into structures that require energy to be maintained and reserves that do not. DEB model was successfully used to investigate the influence of food availability, climate changes, and toxic substances on different species, modeling population dynamics, or organizing principles for metabolism (Jager 2020; Jager et al. 2016; Kearney 2021; Marques et al. 2018).

*Escherichia coli* is a Gram-negative, rod-shaped bacteria that is capable of conducting aerobic respiration in the presence of oxygen and fermentation when oxygen is absent. We choose this species as its morphology, physiology, and genome are well-studied and it plays a huge role in biotechnology and industrial microbiology as a model and host organism (Baniasad and Amoozgar 2015; Blount 2015). It is used in the industry for the production of for instance insulin, erythropoietin, and other recombinant therapeutic proteins as well as biofuels and industrial chemicals like phenol or mannitol (Blount 2015).

## Materials and methods

### Model derivation

Dynamic Energy Budget model is an energy balance that can be formulated for any living organism (Kooijman 2010). The model can be classified as a structured one because the biomass of modeled organisms can be divided into two main groups of compartments: structures, which require energy to maintain, and reserves which do not. According to the model, an organism obtains energy from the environment through its surface and stores it in reserves. Assimilated energy can then be used for different purposes. A fixed fraction *κ* of mobilized energy is used with priority for somatic maintenance and for the increase in the mass of structures that define growth in the DEB context. Maturation, maturity maintenance, and reproduction are powered by the remaining fraction of mobilized energy (1 − *κ*) (Kooijman 2010). The reserves play an important role in DEB models as they enable to include metabolic memory, to smooth out fluctuations in substrate availability, and capture changes in the chemical composition of organisms (Kooijman 2010). Moreover, it is possible to build different DEB models with multiple reserves and structures when it is needed. More details about the DEB model and theory can be found in Kooijman (2010).

In terms of DEB theory, the bacteria culture in a liquid medium can be modeled as one organism. It means that state variables like volume, mass of structures, and mass of reserves of single bacteria can be simply added to each other to obtain values for the whole population living in the bioreactor. Such model organism grows by cell divisions, increasing its volume and surface area proportionally to the number of cells. Therefore, it has to be considered a V1-morph, an organism which surface area is proportional to its volume during growth (Grossowicz et al. 2017; Kooijman 2010; Livanou et al. 2019; Lorena et al. 2010). Some physiological processes like nutrient uptake are proportional to the surface area of the organism, and others like maintenance costs are proportional to the volume of the organism (Kooijman 2010). The ratio between surface area and volume has a huge influence on the organism’s metabolism (Kearney 2021; Kooijman 2010).

**Fig. 1:**
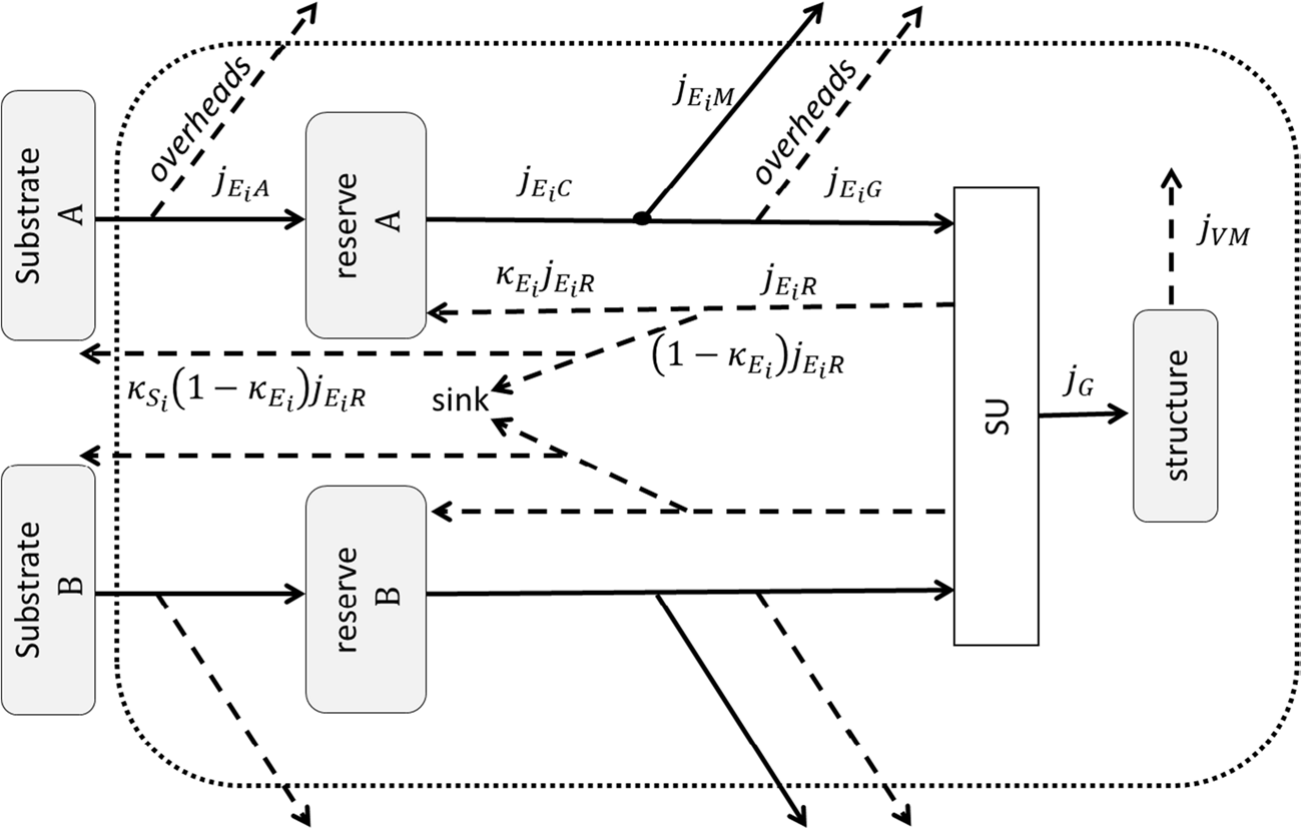
Scheme of DEB model for microorganism population with two reserves and one structure. The dotted line represents the cell surface, and the arrows represent mass fluxes and are described by the appropriate symbols (only for substrate A and reserve A path, as the path for B can be described analogously). SU stands for the synthesizing unit

In the present study, we conducted the bioprocess with a sole carbon source of glucose and a sole nitrogen source of NH_4_^+^. In that case, the carbon and nitrogen assimilation pathways in *E. coli* are basically independent (Kim and Gadd 2019; Reitzer 2003; Willey et al. 2020). The carbon is assimilated through glycolysis and the citric acid cycle and the nitrogen through the reductive amination pathway or glutamine synthetase-glutamate synthetase system (Kim and Gadd 2019; van Heeswijk et al. 2013; Willey et al. 2020). The strong homeostasis assumption in DEB theory implies that the chemical composition of the reserve or structure does not change in living organisms (Kooijman 2010). Therefore, to model two or more independent assimilation pathways, we need to consider the multi-reserve system, with distinct reserves for each pathway. We assume that the assimilation of substrates is conducted directly from the culture medium, which simplified the model by omitting the feeding rate (Grossowicz et al. 2017; Kooijman 2010; Livanou et al. 2019; Lorena et al. 2010). The scheme of the model is presented in Fig. 1, and the symbols of parameters with units are listed in Table 1; fluxes are given in C moles or moles of certain substances per hour.

**Table 1:** Symbols of DEB model and Monod’s model parameters with their estimated or fixed values

Symbol	Description	Value	Unit
*DEB model*			
${j}_{{E}_{C}Am}$	Max. specific C substrate assimilation rate	1.7396*	C-mol_EC_ C-mol_MV_^−1^ h^−1^
${j}_{{E}_{N}Am}$	Max. specific N substrate assimilation rate	0.2405*	mol_EN_ C-mol_MV_^−1^ h^−1^
${K}_{C}$	Half saturation concentration for C	10^−6^	C-mol_SC_ L^−1^
${K}_{N}$	Half saturation concentration for N	10^−6^	mol_SN_ L^−1^
${\dot{k}}_{E}$	Reserve turnover rate	5.3**	h^−1^
${j}_{{E}_{C}M}$	Specific maintenance cost paid by C reserve	0.6104*	C-mol_EC_ C-mol_MV_^−1^ h^−1^
${j}_{{E}_{N}M}$	Specific maintenance cost paid by N reserve	0.0010*	mol_EN_ C-mol_MV_^−1^ h^−1^
${y}_{{E}_{C}V}$	Yield factor of C reserve to structure	1.1	C-mol_EC_ C-mol_MV_^−1^
${y}_{{E}_{N}V}$	Yield factor of N reserve to structure	0.23	mol_EN_ C-mol_MV_^−1^
${y}_{{SE}_{C}}$	Yield factor of C substrate to C reserve	1.1	C-mol_SC_ C-mol_EC_^−1^
${y}_{{SE}_{N}}$	Yield factor of N substrate to N reserve	1.05	mol_SN_ C-mol_EN_^−1^
${\kappa }_{{E}_{C,N}}$	Fraction of rejected flux incorporated in reserves	0.9	–
${\kappa }_{{S}_{C,N}}$	Fraction of rejected flux turned back to substrates	1	–
${j}_{{VM}_{i}}$	Specific maintenance cost paid by structure	0	C-mol_MV_ C-mol_MV_^−1^ h^−1^
*Monod’s model*			
${\mu }_{max}$	Maximum specific growth rate	0.51*	h^−1^
${Y}_{X{S}_{C}}$	Yield of biomass on C substrate	0.45*	C-mol_X_ C-mol_SC_^−1^
${Y}_{X{S}_{N}}$	Yield of biomass on N substrate	4.74*	C-mol_X_ mol_SN_^−1^
${K}_{C}$	Half saturation concentration for C	10^−6^	C-mol_SC_ L^−1^
${K}_{N}$	Half saturation concentration for N	10^−6^	mol_SN_ L^−1^

^*^Estimated simultaneously; **the mean value from C and N limitation separate estimates; see SI for more details

The specific assimilation flux ${j}_{{E}_{i}A}$ of substrates C and N are given by Monod-type equation:

1 $${j}_{{E}_{i}A}={j}_{{E}_{i}Am}\frac{{S}_{i}}{{K}_{i}+{S}_{i}}$$

where $i$ is the index denotes substrate C or N; ${j}_{{E}_{i}Am}$ is the maximum specific assimilation flux, ${S}_{i}$ is substrate concentration (C or N) [C-mol_SC_ L^−1^] or [mol_SN_ L^−1^]; ${K}_{i}$ is the half saturation concentration for C or N. Assimilated substrates are stored in reserves. The reserves are mobilized, which is represented by the mobilization flux ${j}_{{E}_{i}C}$. Mobilization specific flux is proportional to the reserves density ${m}_{{E}_{i}}$ in [C-mol_EC_ mol_V_^−1^] or [mol_EN_ mol_V_^−1^]:

2 $${j}_{{E}_{i}C}={m}_{{E}_{i}}\left({\dot{k}}_{E}-\dot{r}\right)$$

where ${\dot{k}}_{E}$ is the reserve turnover rate; $\dot{r}$ is the specific growth rate $\frac{1}{{M}_{V}}\frac{d{M}_{V}}{dt}$ where ${M}_{V}$ is structural mass in [C-mol_MV_]. The first-order dynamics follow directly from the assumptions of DEB theory (Kooijman 2010). Somatic and maturity maintenance ${j}_{{E}_{i}M}$ are paid from mobilization flux ${j}_{{E}_{i}C}$. The remaining part of ${j}_{{E}_{i}C}$ goes to growth flux ${j}_{{E}_{i}G}$; therefore, it can be expressed as:

3 $${j}_{{E}_{i}G}= {j}_{{E}_{i}C}-{j}_{{E}_{i}M}$$

The maturation process is not modeled in the case of microorganisms (Grossowicz et al. 2017; Kooijman 2010; Livanou et al. 2019; Lorena et al. 2010). The synthesizing unit SU is responsible for integrating growth fluxes from different reserves, in this study from C reserves and N reserves.

The specific growth fluxes ${j}_{{E}_{i}G}$ are parallel and complementary and they interact with each other in SU to create specific growth flux ${j}_{G}$ (Grossowicz et al. 2017; Kooijman 2010; Livanou et al. 2019; Lorena et al. 2010):

4 $${j}_{G}={\left[{{j}_{P}}^{-1}+\sum_{i\in (C,N)}{\left(\frac{{j}_{{E}_{i}G}}{{y}_{{E}_{i}V}}\right)}^{-1}-{\left(\sum\nolimits_{i\in (C,N)}\frac{{j}_{{E}_{i}G}}{{y}_{{E}_{i}V}}\right)}^{-1}\right]}^{-1}$$

For more details, please see Supporting Information (Fig. S9). Assuming that the specific rate of product formation ${j}_{P}$ is much larger than the fluxes entering the SU, the equation can be simplified:

5 $${j}_{G}={\left[\sum\nolimits_{i\in (C,N)}{\left(\frac{{j}_{{E}_{i}G}}{{y}_{{E}_{i}V}}\right)}^{-1}-{\left(\sum\nolimits_{i\in (C,N)}\frac{{j}_{{E}_{i}G}}{{y}_{{E}_{i}V}}\right)}^{-1}\right]}^{-1}$$

The ${y}_{{E}_{i}V}$ represents the yield coefficient or conversion factor used to convert reserves flux ${j}_{{E}_{i}G}$ into corresponding structures flux ${j}_{G}$ and vice versa. The chemical composition of structures cannot be changed; therefore, an excess of ${j}_{{E}_{i}G}$ is rejected as ${j}_{{E}_{i}R}$ which can be partially returned into reserves ${{\kappa }_{{E}_{i}}j}_{{E}_{i}R}$, or excreted from the cells ${\left({1-\kappa }_{{E}_{i}}\right)j}_{{E}_{i}R}$. The rejected flux ${j}_{{E}_{i}R}$ can be thus expressed as:

6 $${j}_{{E}_{i}R}={j}_{{E}_{i}G}-{y}_{{E}_{i}V}{j}_{G}$$

The structures can be used to pay maintenance costs in case when ${j}_{{E}_{i}C}$ is insufficient. Each of the component fluxes can be expressed by the Switch Model (Grossowicz et al. 2017; Kooijman 2010; Livanou et al. 2019; Lorena et al. 2010):

7 $${j}_{{VM}_{i}}=\left({j}_{{E}_{i}M}-min\left({j}_{{E}_{i}C},{j}_{{E}_{i}M}\right)\right){{y}_{{E}_{i}V}}^{-1}$$

and the general ${j}_{VM}$ flux as:

8 $${j}_{VM}=\sum\nolimits_{i\in (C,N)}{j}_{{VM}_{i}}$$

The specific growth rate is simply the difference between ${j}_{G}$ and ${j}_{VM}$:

9 $$\dot{r}={j}_{G}-{j}_{VM}$$

However, in this study, we assume a negligible decrease in structure and therefore $\dot{r}={j}_{G}$. The mass balance for substrates ${S}_{i}$, reserves ${m}_{{E}_{i}},$ and structure ${M}_{V}$ is given by the set of equation:

10 $$\frac{d}{dt}{S}_{i}=\left[-{j}_{{E}_{i}A}+{{\kappa }_{{S}_{i}}\left({1-\kappa }_{{E}_{i}}\right)j}_{{E}_{i}R}\right]{{y}_{{SE}_{i}}\frac{1}{V}M}_{V}+\frac{1}{V}{\dot{J}}_{{NH}_{3}}(only\;for\;{S}_{N})$$

11 $$\frac{d}{dt}{m}_{{E}_{i}}={j}_{{E}_{i}A}-{j}_{{E}_{i}C}+{{\kappa }_{{E}_{i}}j}_{{E}_{i}R}-\dot{r}{m}_{{E}_{i}}$$

12 $$\frac{1}{V}\frac{d}{dt}{M}_{V}=\dot{r}\frac{1}{V}{M}_{V}$$

where $V$ denotes the volume of growth medium in the bioreactor (here $V$ = 1L). Moreover, ammonia can be produced as a metabolite (overheads of assimilation and growth, dissipation) and excreted from the cell increasing the total concentration of ammonia in the medium. It has to be included in mass balance and calculations by introducing ${\dot{J}}_{{NH}_{3}}$ [mol h^−1^] which is the total flux of metabolite ${\text{NH}}_{3}$ (see SI for more details about mass balance).

To eliminate the unphysical model solution, two constraints were defined. First, if ${j}_{{E}_{i}C}<{j}_{{E}_{i}M}$, the maintenance is not paid and the organism dies. The death means that all metabolic fluxes are equal 0, the substrates are not being uptake anymore, minerals are not produced, the reserves are not mobilized, and the growth does not continue. We assume that up to a few hours after death the bacterial cells do not undergo destruction and can be detected by our analytical methods. Note that maintenance can also be filled from structures; however, in this study, we assumed that ${j}_{VM}$ = 0 mainly because we did not obtain sufficient experimental evidence of this process. Second, if the concentration of substrate $i$ is equal to or lower than 0, the assimilation flux ${j}_{{E}_{i}A}$ is also equal to 0. This constraint prevents solving the model equations for unrealistic negative values of substrate concentrations, and is important in the case of cell transfer to limitation media (Chapter 3 in SI).

### Monod’s equation

The derived DEB model was compared to often-used growth kinetics given by the set of three equations:

13 $$\frac{dX}{dt}=\mu X$$

14 $$\frac{d{S}_{C}}{dt}=-\frac{1}{{Y}_{X{S}_{C}}}\mu X$$

15 $$\frac{d{S}_{N}}{dt}=-\frac{1}{{Y}_{X{S}_{N}}}\mu X$$

where $X$ is biomass concentration [C-mol L^−1^], and ${S}_{C}$ and ${S}_{N}$ carbon and nitrogen substrate concentration in the medium [C-mol L^−1^] or [mol L^−1^]. Note that the yields of biomass production for C and N substrates ${Y}_{X{S}_{C}}$ and ${Y}_{X{S}_{N}}$ are assumed to be constant during the process. The specific growth rate $\mu$ was given by the Monod equation (Monod 1949) with an extension to two limiting substrates (Zinn et al. 2004):

16 $$\mu = {\mu }_{max}\frac{{S}_{C}}{{K}_{S}+{S}_{C}}\frac{{S}_{N}}{{K}_{N}+{S}_{N}}$$

where ${K}_{S}$ and ${K}_{N}$ are half saturation constants for C and N substrates, respectively.

### Growth kinetics

*Escherichia coli* strain (PCM 2057) was obtained from Ludwig Hirschfeld Institute of Immunology and Experimental Therapy-Polish Academy of Science. *E. coli* cultures were prepared in sterile Erlenmeyer flasks by transferring 1 ml of inoculum (see SI) to 100 ml of appropriately modified M9 liquid medium (Tab. S1 and S2).

In order to determine the growth kinetics in carbon-limiting conditions, three media were prepared (Tab. S2). The concentration of NH_4_Cl was 0.075 gL^−1^ (150% of NH_4_Cl concentration in the basic medium), while the concentration of glucose was reduced to 25, 50, and 75% of the basic concentration (1, 2, and 3 gL^−1^, respectively). Such conditions provide unlimited access to NH_4_Cl with the simultaneous depletion of glucose after some time of culturing.

In the case of experiments determining growth limitation by nitrogen, the basic concentration of glucose was increased by 50% to ensure its excess, while the concentration of NH_4_Cl was reduced to 0.075, 0.125, and 0.25 gL^−1^ (15, 25, and 50% of NH_4_Cl basic concentration) (Tab. S2). The concentration of NH_4_Cl was lowered more than the concentration of glucose because nitrogen limitation is less severe for the bacteria and growth stops much later.

The cultures were incubated at 37 °C on a rotary shaker (at 160 rpm, IKA KS 4000, Germany). The samples were collected in triplication, every hour while the culture was being grown. The concentrations of bacteria cells (optical density at 550 nm), glucose concentration (enzymatic test, Biomaxima, Tab. S3), and nitrogen concentration (colorimetric cuvette test, Spectroquant, Merck) were measured in each sample (please see SI for details, Fig. S1-S5).

The batch culture was chosen mainly because most industrial bioprocesses are conducted in fed-batch not in continuous reactors (Luo et al. 2021). Moreover, the control and optimization of batch processes using mechanistic models seems to be one of the challenges of modern industrial microbiology (Luo et al. 2021; Rathore et al. 2021).

### Shock limitation

In order to determine shock limitation, the basic M9 medium (4 gL^−1^ glucose and 0.05 gL^−1^ NH_4_Cl) was inoculated and cultured as described above (section “Growth kinetics”) for 4 h. The concentrations of bacteria cells, glucose, and nitrogen were controlled. The culturing was sopped in the mid-log growth phase, the bacterial suspensions were centrifuged (Hettich Universal 320R, 4 min, 4000 rpm), and the cell pellet was separated from the supernatant. The cells were transferred to two different fresh (sterile) mediums. 

The first flask consisted of 100 mL of basic M9 medium deprived of glucose and the second one was without NH_4_Cl. The whole transfer procedure lasted app. 30 min. Then, the cultures were again incubated at 37 °C with shaking at 160 rpm on a rotary shaker. The samples were collected in triplication every 30 min. As previously, the concentrations of bacteria cells, glucose, and nitrogen were measured.

### Biomass composition

C, H, N, and S content was determined separately in cultures where growth was stopped due to lack of carbon or nitrogen source. The C-limited culture was conducted in an M9 medium with glucose and NH_4_Cl in initial concentrations of 1.0 g L^−1^ and 0.75 gL^−1^, respectively. The N-limited culture was prepared analogically with the initial concentration of glucose 6 gL^−1^ and NH_4_Cl 0.075 gL^−1^. The medium was inoculated and incubated for 8 h in previously described conditions (section “Growth kinetics”). After this time, when the logarithmic growth phase was finished and the stationary phase began, the culturing was stopped. The bacterial suspensions were centrifuged (Hettich Universal 320R, 20 min, 9000 rpm), and the pellet was separated from the supernatant and dried for 24 h (Memmert, 45 °C). The content of C, H, N, and S in dry bacterial pellets was determined by CHNS Elemental Analyzer Vario EL Cube, with acetanilide as a standard (in the external laboratory). The content of C, H, and N in biomass was used to determine the molecular weights and elemental composition of structure and biomass in different limitations conditions. The stoichiometry of C-limited and N-limited biomass was used to calculate the reserves densities in both cases. See Supporting Information for details.

### Parameter estimation

The DEB model parameters (${j}_{{E}_{i}Am}$, ${j}_{{E}_{i}M}$) and Monod’s model parameters (${\mu }_{max}$, ${Y}_{X{S}_{C}}$, and ${Y}_{X{S}_{N}}$) were fitted simultaneously to all datasets containing time-depending mean values of biomass, glucose, and ammonium concentrations obtained for different C and N limitation conditions (see more details in SI). The reserve turnover rate ${\dot{k}}_{E}$ was estimated separately from C and N limitations data, and its mean value was used (see SI). The weighted nonlinear least-square method was used. The loss function, weighted sum of squared errors (WSSE), for three different variables biomass ${X}_{j}$, C substrate concentration ${S}_{Cj}$, and N substrate concentration ${S}_{Nj}$ in six different limitation scenarios ($j$ = 1,2,…,6) was given by the equation:

$$WSSE=\sum_{j=1}^{m}\left({W}_{Xj}\sum_{i=1}^{n}{\left[{X}_{j}\left({t}_{i},\mathbf{p}\right)-{Y}_{Xij}\right]}^{2}+{W}_{{S}_{C}j}\sum_{i=1}^{n}{\left[{S}_{Cj}\left({t}_{i},\mathbf{p}\right)-{Y}_{{S}_{C}j}\right]}^{2}+{W}_{{S}_{N}j}\sum_{i=1}^{n}{\left[{S}_{Nj}\left({t}_{i},\mathbf{p}\right)-{Y}_{{S}_{N}j}\right]}^{2}\right)$$

where $n$ is the number of data points for each variable in each scenario (here unchanged between scenarios); ${Y}_{Xij}$, ${Y}_{{S}_{C}j}$, and ${Y}_{{S}_{N}j}$ represent data points for $i$ th time and $j$ th scenario; ${W}_{Xj}$, ${W}_{{S}_{C}j}$, and ${W}_{{S}_{N}j}$ are the weight coefficients for each variable dataset in each limitation scenario. The weight coefficient was defined as a reciprocal of mean variance of measured values of one variable in a certain limitation scenario.

$${W}_{j}= {\left(\frac{\sum_{i=1}^{n}{{s}^{2}}_{ij}}{n}\right)}^{-1}$$

Note that the variance ${{s}^{2}}_{ij}$ is calculated for each variable at each time of incubation as they were measured in three repetitions. The loss function was minimized using the Nelder-Mead Simplex Method in Matlab (Lagarias et al. 1998). The set of DEB model and Monod’s model differential equations were solved using the Runge–Kutta method in each step of loss function minimization. The goodness of fit was described by the adjusted coefficient of determination (${{R}^{2}}_{adj}$), and root mean square error (RMSE). More details about the parameter values estimation procedure can be found in SI.

## Results

**Fig. 2:**
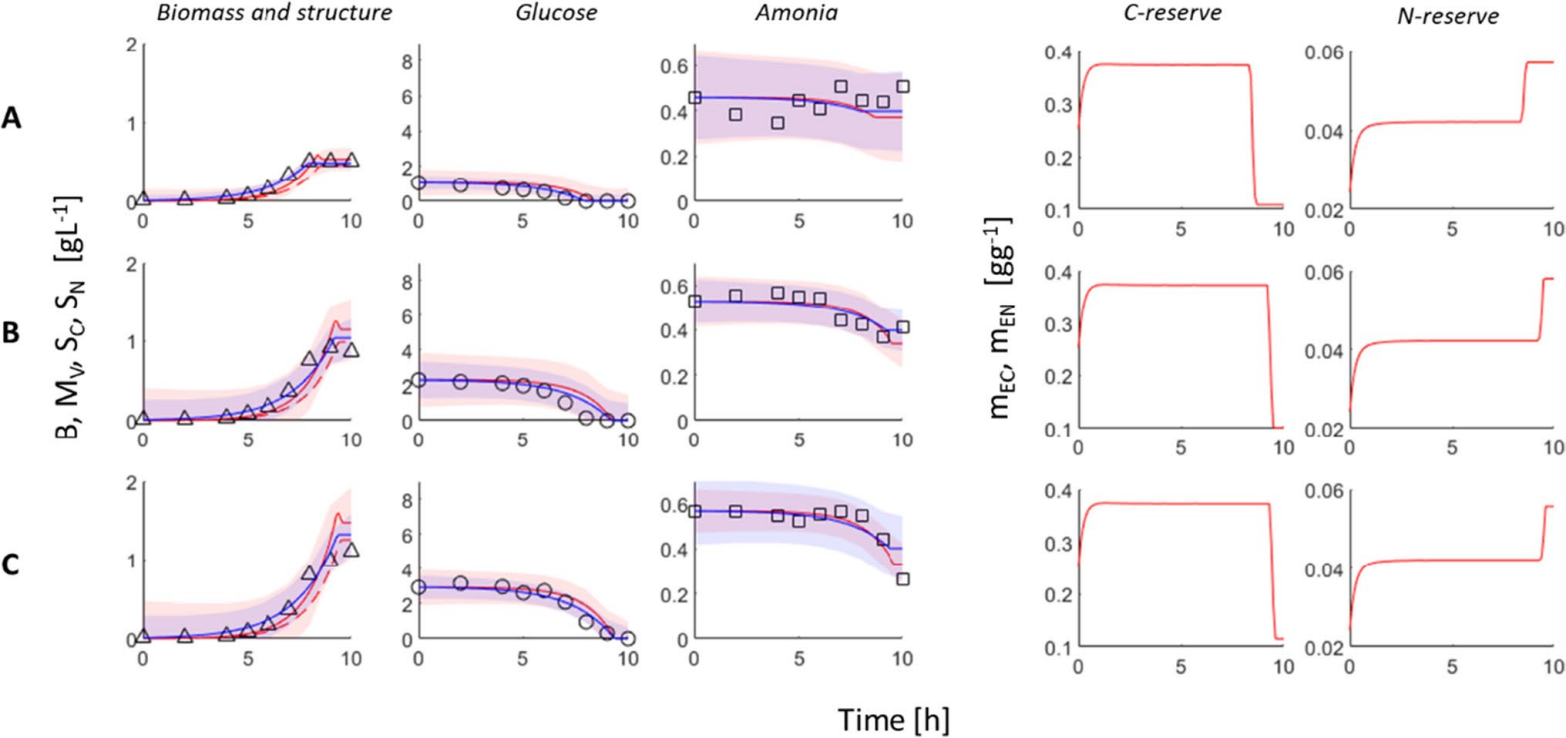
DEB and Monod’s model fit to experimental data obtained in three different C limitation conditions. Rows A, B, and C correspond to different initial nominal concentration of glucose 1, 2, and 3 gL^−1^, respectively. Triangles, circles, and squares represent datapoints for each variable, biomass, C substrate, and N substrate concentrations. Solid red lines indicate main DEB model variables, dashed red line indicates structure, blue lines Monod’s model. Red and blue area represents 95% nonsimultaneous prediction interval for the next observation

**Fig. 3:**
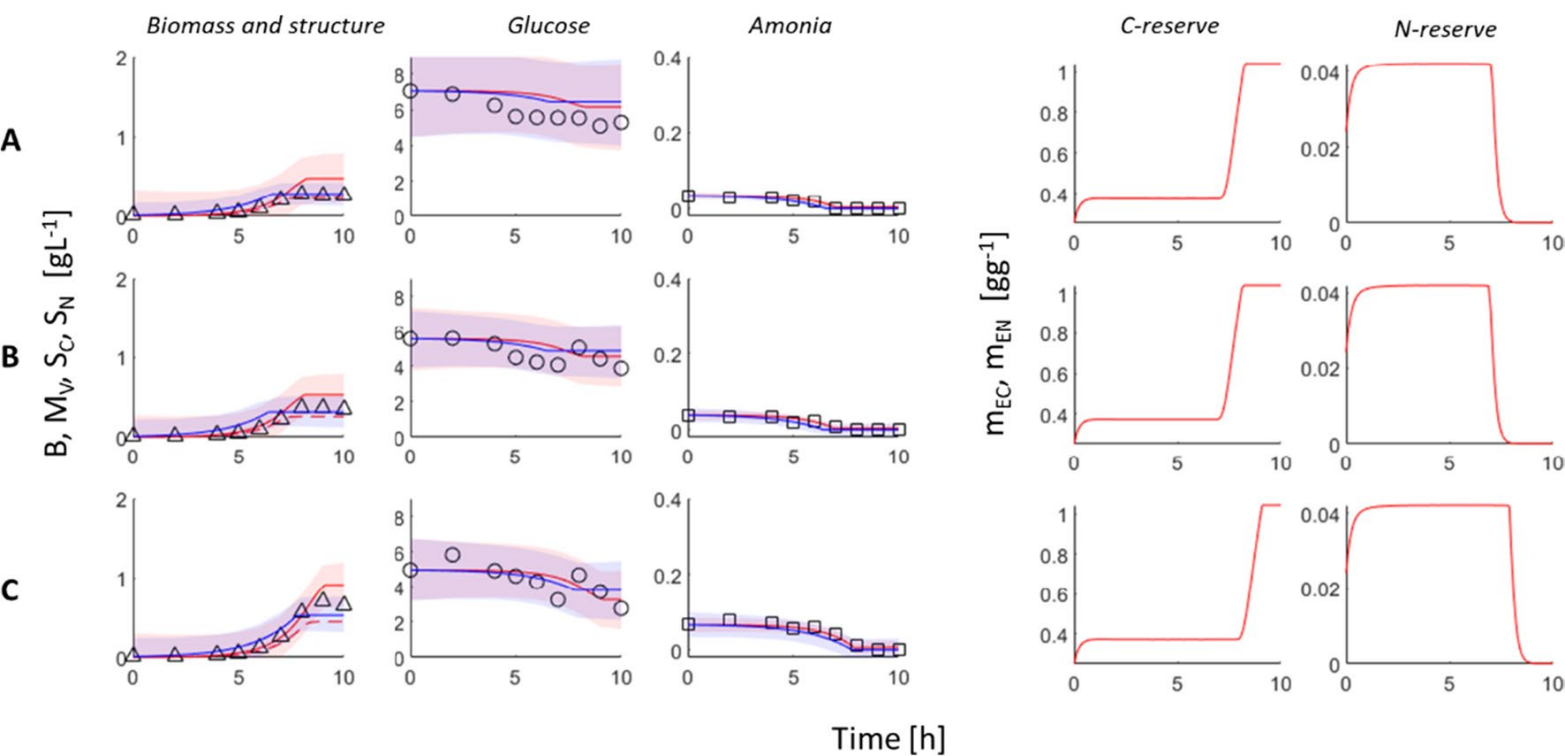
DEB and Monod’s model fit to experimental data obtained in three different C limitation conditions. Rows A, B, and C correspond to different initial nominal concentration of NH_4_Cl 0.075, 0.125, and 0.25 gL^−1^, respectively. Triangles, circles, and squares represent datapoints for each variable, biomass, C substrate, and N substrate concentrations. Solid red lines indicate main DEB model variables, dashed red line indicates structure, blue lines Monod’s model. Red and blue area represents 95% nonsimultaneous prediction interval for the next observation

The growth of *E. coli* in different substrates limitations conditions along with DEB and Monod’s models fits to experimental data are presentment in Figs. 2 and 3. In the first three columns, the changes in biomass, glucose, and ammonia concentrations are shown, in the last two columns the changes in C and N reserve densities.

The growth limitation by carbon substrate was tested in three scenarios with different low initial glucose concentrations (1, 2, and 3 gL^−1^). By looking only at experimental data, it can be observed that bacteria growth stops approximately at the same time when glucose is completely used (Fig. 2). It happens after 8–10 h depending on initial glucose concentrations. A low decrease in ammonia concentration was noted in the last hours of incubation. The growth inhibition by ammonia can be described analogically. Initial NH_4_Cl concentrations were 0.075, 0.125, and 0.25 gL^−1^. The growth is finished approximately when ammonia concentration decreases to 0 (Fig. 3). The decrease in glucose concentrations can be observed in the whole incubation interval of 0–10 h.

Both models were fitted simultaneously to six limitation scenarios (Figs. 2 and 3). The goodness of fit was slightly better in the case of Monod’s model (${{R}^{2}}_{adj}$ = 0.58, RMSE = 0.3232) compared to the DEB model (${{R}^{2}}_{adj}$ = 0.49, RMSE = 0.3574). The estimates of model parameter values are given in Table 1. In the presented setup, the DEB model has more degrees of freedom than Monod’s model, even though the DEB model seems to be more constrained by thermodynamics and by undertaken assumptions which results in a slightly worse fit but better mechanistic explanation of observed growth patterns.

The biochemistry of glucose and ammonia in *E. coli* has a great impact on the final form of the derived DEB model. Both substrates are assimilated and metabolized in known but different biochemical pathways. The different assimilation and metabolism of glucose and ammonia implicate the use of two distinct reserves and assimilation paths in the DEB model. The carbon and nitrogen fluxes are bound together in a synthesizing unit, to create a new structure (Fig. 1). Glucose can be used by *E. coli* as a source of energy and a source of building blocks for different cellular structures. Glucose is catabolized to pyruvate through glycolysis, and then Acetyl-CoA fuels the tricarboxylic acid (TCA) cycle, where electron donors such as NADH and FADH_2_ are produced and used in the electron transport chain to generate proton motive force for ATP synthesis via oxidative phosphorylation. The precursor metabolites are intermediates of glycolysis and the TCA cycle. For instance, glucose-6-phosphate is used for nucleotides and carbohydrates synthesis, pyruvate for amino acid synthesis, acetyl-CoA for lipids, and α-ketoglutarate for glutamate and amino acid synthesis (Kim and Gadd 2019; Willey et al. 2020). In the case of *E. coli*, the ammonia nitrogen is not used as a source of energy. Nitrogen is an important component of proteins, nucleic acids, and coenzymes. Ammonia nitrogen is more reduced than any other form of nitrogen and therefore it is the preferred source of nitrogen for microorganisms which results in the highest growth rates (Kim and Gadd 2019; van Heeswijk et al. 2013; Willey et al. 2020). It is assimilated in two different pathways depending on its concentration in the environment. When the ammonia concentration is high, glutamate is formed from α-ketoglutarate in the reductive amination pathway. If the concentration of ammonia is low, some bacteria can use glutamine synthetase-glutamate synthetase system to assimilate it. In both cases, the glutamate is formed which is an amino donor for further reactions (Kim and Gadd 2019; van Heeswijk et al. 2013; Willey et al. 2020).

It can be observed that both models predict exponential growth of bacteria which is stooped by the lack of one limiting substrate in the case of Monod’s model, or by the decrease of limiting reserves density below the critical value, so the maintenance cannot be paid anymore, in case of DEB model. According to the DEB model, bacteria can live and maintain biochemical transformations and even grow in case of lack of substrate until the reserves are able to fill all these energetic needs (Kooijman 2010). Moreover, if reserve density decreases under critical value which can be equivalent to the death of a microorganism, there is a possibility to mobilize the structure and maintain it even longer (Grossowicz et al. 2017; Livanou et al. 2019; Lorena et al. 2010). These features of bacteria cultivation were experimentally confirmed previously (Williams 1967). However, in this study, the small decrease in the biomass of *E. coli* at the end of the batch process (Fig. 2) can be explained by the decrease in C reserve density and a small increase in structures. The decrease in biomass caused by the decrease in N reserve was negligibly small (Fig. 3).

The results of shock limitation experiments showed that biomass and substrate concentration did not change after bacteria were transferred to the medium without one of the substrates. The lack of changes can indicate a lack or negligibly small metabolic activity or death. The observations agree with Monod’s model where the lack of one of the limiting substrates stops growth, and with the DEB model in the case when limiting reserves are quickly (up to 30 min) used (Fig. S6), which confirms high reserve turnover rate estimates (Table 1). The concentration of biomass of microorganism does not decrease up to 6.5 h after the transfer, which means that decrease confirms our assumptions that shrining is not observed in this time interval (${j}_{VM}$ = 0), and/or cells do not undergo destruction after death (or the method used to measure cell concentration is insensitive for mentioned changes).

Maximum specific assimilation rate ${j}_{{E}_{i}Am}$ was higher for glucose (1.7396 C-mol_EC_ C-mol_MV_^−1^ h^−1^) than for ammonia (0.2405 mol_EN_ C-mol_MV_^−1^ h^−1^), which is related to the general demand for these substrates. The value of ${j}_{{E}_{N}Am}$ was higher than values obtained for cyanobacterium *Prochlorococcus marinus*, green algae, and phytoplankton 0.0054 mol NH_4_ C-mol_MV_^−1^ h^−1^ (in the original unit: 0.13 mol NH_4_ C-mol_MV_^−1^ day^−1^) (Grossowicz et al. 2017; Marañón et al. 2013), 0.042 mol NH_4_ C-mol_MV_^−1^ h^−1^ (1.0 mol NH_4_ C-mol_MV_^−1^ day^−1^) (Geider et al. 1998; Lorena et al. 2010), and 0.0058 mol NH_4_ C-mol_MV_^−1^ h^−1^ (0.14 mol NH_4_ C-mol_MV_^−1^ day^−1^) (Livanou et al. 2019), respectively. It can be explained by the faster growth of *E. coli*, and therefore faster substrate usage in bioreactors compared to the growth of previously studied microorganisms. The carbon assimilation fluxes in the mentioned studies were determined for photosynthesizing microorganisms and therefore they were not compared with the results obtained in this study.

The half-saturation constants (${K}_{C}$ and ${K}_{N}$) for both models were fixed to 10^−6^ mol L^−1^. The assumed values were close to values used in other studies; for photosynthesizing microorganisms, ${K}_{C}$ was in the range 3.2–233 µmol L^−1^, and ${K}_{N}$ in the range 0.11–0.40 µmol L^−1^ (Grossowicz et al. 2017; Livanou et al. 2019; Lorena et al. 2010). Assumed values were substantially lower than the concentrations of glucose and ammonia used. In that case, the assimilation flux ${j}_{{E}_{i}A}$ (Eq. (1)) does not depend on substrate concentration ($\frac{{S}_{i}}{{K}_{i}+{S}_{i}} \approx 1)$ and is equal to ${j}_{{E}_{i}Am}$. The substrate concentration ${S}_{i}$ can influence ${j}_{{E}_{i}A}$ only when it decreases to very low values, close to 10^−6^ mol L^−1^.

The estimated reserve turnover rate of 5.3 h^−1^ was higher than previously found values for microorganisms which were in the range 0.082–0.22 h^−1^ (1.96–5.2 day^−1^) (Grossowicz et al. 2017; Livanou et al. 2019; Lorena et al. 2010). The specific maintenance cost ${j}_{{E}_{i}M}$ were equal to 0.6104 C-mol_EC_ C-mol_MV_^−1^ h^−1^ and 0.0010 mol_EN_ C-mol_MV_^−1^ h^−1^ for C and N reserves, respectively. ${j}_{{E}_{C}M}$ were much higher, and ${j}_{{E}_{N}M}$ only slightly higher than values used in case of phytoplankton: 0.00042 mol_EN_ C-mol_MV_^−1^ h^−1^ (0.01 mol_EN_ C-mol_MV_^−1^ day^−1^) for C and N reserves (Livanou et al. 2019), and other microorganisms: 0.0023 mol_EN_ C-mol_MV_^−1^ h^−1^ (0.054 mol_EN_ C-mol_MV_^−1^ day^−1^) for C reserve, and 0.0005 mol_EN_ C-mol_MV_^−1^ h^−1^ (0.012 mol_EN_ C-mol_MV_^−1^ day^−1^) for N reserve (Grossowicz et al. 2017; Lorena et al. 2010). High reserve turnover rate and high maintenance flux testify to the intensity in which metabolic transformations take place in *E. coli*.

The low value of ${j}_{{E}_{N}M}$ indicates the lower need for ammonia. It is not used as an energy source; however, it can be used to pay maintenance costs as a substrate to repair existing structure (Kooijman 2010).

The fraction of rejected flux incorporated in reserves ${\kappa }_{{E}_{C,N}}$ was assumed to be equal 0.9, and the fraction of rejected flux turned back to substrates ${\kappa }_{{S}_{C,N}}$ was assumed to be equal 1 (Table 1). Different values were used in previous studies, for ${\kappa }_{{E}_{C,N}}$ 0.5 (Grossowicz et al. 2017), 0.95 (Livanou et al. 2019), and 0.7 (Lorena et al. 2010), and for ${\kappa }_{S}$ 0.95 and 0 for C and N respectively (Grossowicz et al. 2017). Assumed values ensure that most of the rejected flux returns to reserves, and the remaining part is totally excreted from the cells in the form of substrate (because ${\kappa }_{{S}_{C,N}}$ = 1). If ${\kappa }_{{S}_{C,N}}$ < 1, the part of rejected flux would go out from the considered system, disappear in the sink (see Fig. 1), or, which is more accurate, be excreted from the cell or accumulated inside it in a different chemical form than substrates or reserves. This has to be described as a new variable with its own dynamics and included in the mass balance. We do not want to introduce a new variable; however, we leave this model topology because a similar was used in a previous study (Grossowicz et al. 2017) and can be a starting point for more complex model derivation.

The stoichiometry of structure C-mol was determined along with biomass elemental composition and molecular weights of structure, reserves, and biomass resulting in the formula $C{H}_{1.96}{O}_{0.45}{N}_{0.22}$ (see SI). The mean value of molecular weight of C-mol of structure ${M}_{W\text{Mv}}$ was equal to 29.76 [g mol^−1^], and was used along with molecular weights of reserves for mass-moles recalculation within the DEB model (see SI). The yield factors of reserves to structure were assumed to be slightly higher than chemical indexes of appropriate elements ${y}_{{E}_{C}V}$ =1.1 and ${y}_{{E}_{N}V}$ = 0.23. It means that the efficiency of the transformation of reserves to structures is less than 100% and some losses are generated (see mass balance in SI, Fig. S7 and S8). Similarly, the yield factors of substrates to reserves were assumed to be equal to 1.1 and 1.05 for C and N substrates, respectively (Table 1). The mean biomass C-mol molecular weight was equal to ${M}_{WB}$ = 30.00 [g mol^−1^] and was used within Monod’s model. The values of estimated maximum specific growth rate ${\mu }_{max}$ and yields of biomass on substrates ${Y}_{X{S}_{C}}$ and ${Y}_{X{S}_{N}}$ were similar to those which can be found in literature (Folsom and Carlson 2015; Pramanik and Keasling 1997).

Both models, the DEB model and Monod’s model, described the data very well (Figs. 2 and 3). It seems that Monod’s model is a sufficient tool for simple and general analysis of *E. coli* growth in a batch bioreactor. However, the DEB model brings more insights about the bioprocess and therefore can be used in more complex cases, in which besides biomass concentration, other intracellular features of microorganisms have to be known. Some differences in both models’ predictions and properties are discussed below.

## Discussion

Contrary to Monod’s model, the DEB model gives the opportunity to simulate and predict the biomass composition. According to the DEB model, the chemical composition of structures can be different than the chemical composition of reserves; however, both are assumed to be constant (Kooijman 2010). Therefore, the changes in biomass composition can be modeled as changes in reserve densities (Kooijman 2010).

The simulations show an increase in reserves densities, attributed to limiting substrate, starting from their initial values at the beginning of the bioprocess (Figs. 2 and 3), then steady state, and decrease when the concentration of substrate in the medium is low and reserves are faster used than filled. The density of the second reserve, not attributed to the limiting substrate, follows a similar pattern at the beginning of the process as previously described (Figs. 2 and 3). However, at the time when the density of the limiting reserve decreases, the density of the second one increases which influences the biomass composition (Tab. S4).

Moreover, it can be shown that the higher the density of limiting reserves in a multi-reserve system (reserves that are not rejected from SU), the higher the growth rate. Many experiments showed that the cell biomass composition is different at different growth rates (Folsom and Carlson 2015; Pramanik and Keasling 1997). For instance, the RNA content in biomass is higher at higher growth rates, and the glycogen, DNA, and protein contents are lower at higher growth rates (Pramanik and Keasling 1997). The energy requirements also change along with growth rates (Pramanik and Keasling 1997). The higher the growth rates, the higher requirements for macromolecules synthesis, polymerization metabolites transport, and maintenance of transmembrane gradients (Pramanik and Keasling 1997). According to DEB theory, RNA belongs to reserves and DNA to structures. Therefore, the DEB theory seems to agree with empirical observation, as well as presented model.

A similar “compartment” approach which captures these observations was proposed by Williams (1967) in his model. The cells were deviated into a synthetic portion fed from nutrient uptake and a structural/genetic portion fed from a synthetic one. The Williams model was used successfully to describe such phenomena as cell size increases in the lag phase and decreases in the stationary phase, the rapid increase of cell number and total biomass in the exponential phase, and changes in chemical composition of cells as a function of growth (Williams 1967). The DEB model compared to the Williams model is more general and can be applied to all living species, not only to microorganisms, and the model equations and its derivation have strong biological foundations and mechanistic explanations (Kooijman 2010).

Moreover, according to the DEB model, the biomass of an organism can be divided into a large number of structures and reserves, which makes the model flexible (Kooijman 2010). However, it has to be pointed out that the higher the number of variables the higher the number of parameters that have to be estimated. The choice of the number and composition of reserves can be related to the purpose of model use; however, it needs a good mechanistical explanation. Here we decide to stick to the reserves related to the two limiting substrates. This allows us to model the changes in the biomass composition throughout the whole process. It should be noted that even though the model describes the process very well in presented form, more reserves and more substrates can be included, for example, the phosphorous substrate and reserve, which may allow us to follow the changes in biomass composition even more precisely.

These model properties can be used to control bioprocesses and increase their efficiencies. The biomass composition can be optimized according to our needs by manipulating with substrate concentrations and growth rates. Results of our studies show that the nitrogen content can be increased from approx. 10.6% up to 11.23% of total biomass by the carbon source limitation (Fig. 2, biomass composition in Tab. S4); similarly, the carbon content can be increased by nitrogen source limitation (Fig. 3, Tab. S4), and the DEB model explains mechanisms of this phenomena. The possibility of manipulation with biomass composition is important in many biotechnologies. For example, phosphorous-rich biomass is desired in biological wastewater treatment plants (Santos et al. 2020). The same biomass of microorganisms rich in N and P can be used as a fertilizer (Spanoghe et al. 2020). Protein-rich biomass of microorganisms can find different applications in the food industry (Choi et al. 2022).

The multi-substrate limitation is a topic worthy of attention which can significantly contribute to improving the efficiency of biotechnological processes. Wierzchowska et al. (2021) indicated that the nitrogen limitation connected with phosphorus one improves the lipid biosynthesis by *Yarrowia lipolytica*. They obtained the highest cellular lipid yield—47.5% (w/w) in the medium where the concentration of KH_2_PO_4_ and (NH_4_)_2_SO_4_ was the lowest. Xin et al. (2010) tested the influence of nitrogen/phosphorus ratio on the *Scenedesmus* sp. LX1 metabolism and the limitation of them as well. They showed that the nitrogen limitation (2.5 mgL^−1^) or phosphorus (0.1 mgL^−1^) can increase lipid accumulation by 30 and 53%, respectively. Patel et al. (2017) proved that the strain *Rhodotorula kratochvilovae* has a higher ability to the lipid accumulation under the nitrogen and phosphorus limitation in comparison to the medium rich of these components. They tested various N and P low concentrations and they indicated the 0.1 gL^−1^ and 0.05 gL^−1^ concentration of N and P, respectively, as the most appropriate conditions. Application of these concentrations results in the highest lipid content—60.3%. All of these examples of bioprocesses can be most probably explained by the presented DEB model and therefore can also be optimized and controlled with the use of this model.

It seems that the DEB model has great potential to model the dynamics of many bioprocesses. Different bioproducts can be associated with different model variables. For example, amino acids such as l-lysine or glutamic acid can be related to the dynamic of structures, glycerol, and polyphosphates to reserves as well as some enzymes like proteases and amylases, and finally bioethanol, biogas, lactic acid, antibiotics, and many other in the DEB context can be attributed to dissipation and overheads of assimilation and growth (Baniasad and Amoozgar 2015; Choi et al. 2022; Nielsen et al. 2022; Shimizu 2008; Weimer et al. 2020). These are only a few examples of popular compounds produced in bioreactors. Production of each of these compounds can be controlled and optimized using the DEB approach.

The changes in biomass composition of *E. coli* growing in carbon and nitrogen limitation conditions were explained by the DEB model, specifically, by the dynamics of C and N reserve densities. It was shown that the DEB model provides the possibility to follow the changes in the microbes’ elemental composition in different growth phases which makes it more informative and explanatory compared to Monod’s model. It describes the dynamics of the changes in the microbes’ composition contrary to the FBA approach which is used to simulate most of the known intracellular metabolites and complex composition of microbes’ biomass for certain growth rates with the assumption of a steady state. Therefore, it seems that the DEB model combines the advantages of the mentioned approaches by including more complexity in cell physiology and keeping the system dynamic and thermodynamically constrained. These features of the presented model can be used for optimization, prediction, and control of different bioprocesses conducted by microorganisms.

## Supplementary Information

Below is the link to the electronic supplementary material.

Supplementary file1 (PDF 558 KB)

## Data Availability

The raw data are available from the corresponding author upon reasonable request.
