# Supplementary material for: Dynamic Energy Budget model for E. coli growth in carbon and nitrogen limitation conditions
Source: Appl Microbiol Biotechnol. 2024 Jul 5;108:408. doi: 10.1007/s00253-024-13245-9 (PMC11226513; doi:10.1007/s00253-024-13245-9)
Supplement: Supplementary file 1 [file 253_2024_13245_MOESM1_ESM.pdf]

Applied Microbiology and Biotechnology

Supporting Information for:

Dynamic Energy Budget model for *E. coli* growth in carbon and nitrogen limitation conditions

Konrad Matyja[1*], Magdalena Lech[1]

[1]Wroclaw University of Science and Technology, Faculty of Chemistry, Department of Micro, Nano, and Bioprocess Engineering, wybrzeże Wyspiańskiego 27, 50-370 Wrocław, Poland

[*]Corresponding Author

Email: konrad.matyja@pwr.edu.pl

Konrad Matyja: ORCID: 0000-0003-1456-1127

Magdalena Lech: ORCID: 0000-0003-1907-2429

Table of Contents

1. Experimental design 3

1.1. Stock solution of *Escherichia coli* culture 3

1.2. Inoculum 3

1.3. Medium 3

1.4. Culturing and sampling 5

1.5. Cells concentration analysis 5

1.6. Glucose analysis 6

1.7. Ammonium analysis 8

2. Biomass composition 10

3. Shock limitation and reserves kinetics 13

4. Mass balance 14

5. Parameters estimation 17

5.1. Units conversion 17

5.2. Estimation algorithm 17

5.3. Estimation procedure 17

5.4. Goodness of fit 18

6. Synthesizing unit 19

References 19

1. Experimental design

1.1. Stock solution of *Escherichia coli* culture

LB medium dedicated to *Escherichia coli* culturing was prepared and sterilized at 121°C, 1 bar per 20 minutes. A single colony of *E. coli* (PCM 2057, Polish Academy of Science, Poland) from the LB plate (containing 2% agar powder (Sigma Aldrich, CAS: 9002-18-0)) was transferred into 100 mL of LB. The flask was incubated at 37°C for 24 hours and shaken at 160 rpm on a rotary shaker (IKA KS 4000, Germany), which led to the formation of a high-density culture. The LB medium with bacteria cells was diluted by sterile glycerol (CAS: 56-81-5, Poch, Poland) in proportion 60:40 (*v:v*) and transferred to the sterile tubes. The tubes were stored at -20ºC.

1.2. Inoculum

The cells from the *E. coli* stock solution were transferred on an LB plate using an inoculation loop and then incubated at 37°C for 24 hours. After this time a single colony of *E. coli* from the LB plate was transferred into 100 mL of sterile LB medium and incubated at 37°C for the next 24 hours and shaken at 160 rpm on a rotary shaker (IKA KS 4000, Germany). The bacteria cultures were in a stationary phase when used for further experiments.

1.3. Medium

To determine the experimental data necessary for model verification, cultures were carried out in the modified M9 medium [https://static.igem.org/mediawiki/2019/2/20/T--Tuebingen--M9_recipe.pdf]. The basic concentrations of mineral salts and glucose are presented in Table S1, and modifications of the M9 medium are described in Table S2.

Table S1. The concentration of components in each cultures of *E.coli* (for 1 basic litter M9 mineral medium)

Component	Concentration of Component	Volume
M9 slat solution (10X)		100 mL
$Na_2HPO_4*2H_20$	58 gL^{-1}	
KH_2PO_4	30 gL^{-1}	
NaCl	5 gL^{-1}	
NH_4Cl	5 gL^{-1}	
Glucose	20 %	20 mL
$MgSO_4$	1 M	1 mL
$CaCl_2$	1 M	0.3 mL
Trace elements		10 mL
EDTA	5 gL^{-1}	
$FeCl_3*6H_2O$	0.83 gL^{-1}	
$ZnCl_2$	84 mgL^{-1}	
$CuCl_2*2H_2O$	13 mgL^{-1}	
$CoCl_2*2H_2O$	10 mgL^{-1}	
H_3BO_3	10 mgL^{-1}	
$MnCl_2*4H_2O$	1.6 mgL^{-1}	
Distilled water	-	867 mL

Table S2. The modified M9 medium (basic concentrations: 4gL^{-1} glucose, 0.5gL^{-1} NH_4Cl)

	Flask No.	Glucose concentration	NH_4Cl concentration
Glucose limitation	1	1 gL^{-1}	0.75 gL^{-1}
	2	2 gL^{-1}	
	3	3 gL^{-1}	
Nitrogen limitation	4	6 gL^{-1}	0.075 gL^{-1}
	5		0.125 gL^{-1}
	6		0.25 gL^{-1}

1.4. Culturing and sampling

To 100 mL of sterile appropriate modified M9 medium 1% (1 mL) of inoculum was introduced. The cultures were incubated at 37°C with shaking at 160 rpm on a rotary shaker (IKA KS 4000, Germany). The samples were collected in triplication while the culture was being grown. The concentrations of bacteria cells (optical density at 500 nm wavelength measurement), glucose, and nitrogen (colorimetric, spectrophotometric measurement) were measured.

1.5. Cells concentration analysis

The cell concentration was determined using the optical density method (OD). OD was measured at 550 nm (Shimadzu, USA) and recalculated into cell concentration using the standard curve given by the equation:

$$C_{E.coli} = 0.3623 A_{550} \quad \text{(S1)}$$

where: $C_{E.coli}$ - is a concentration of dry *E. coli* biomass [gL^{-1}]; A_{550} - is an absorbance at 550 nm [-]. The background absorbance was measured (A_{550} = 0.014) and subtracted from further measurements. The standard curve is shown in Figure S1.

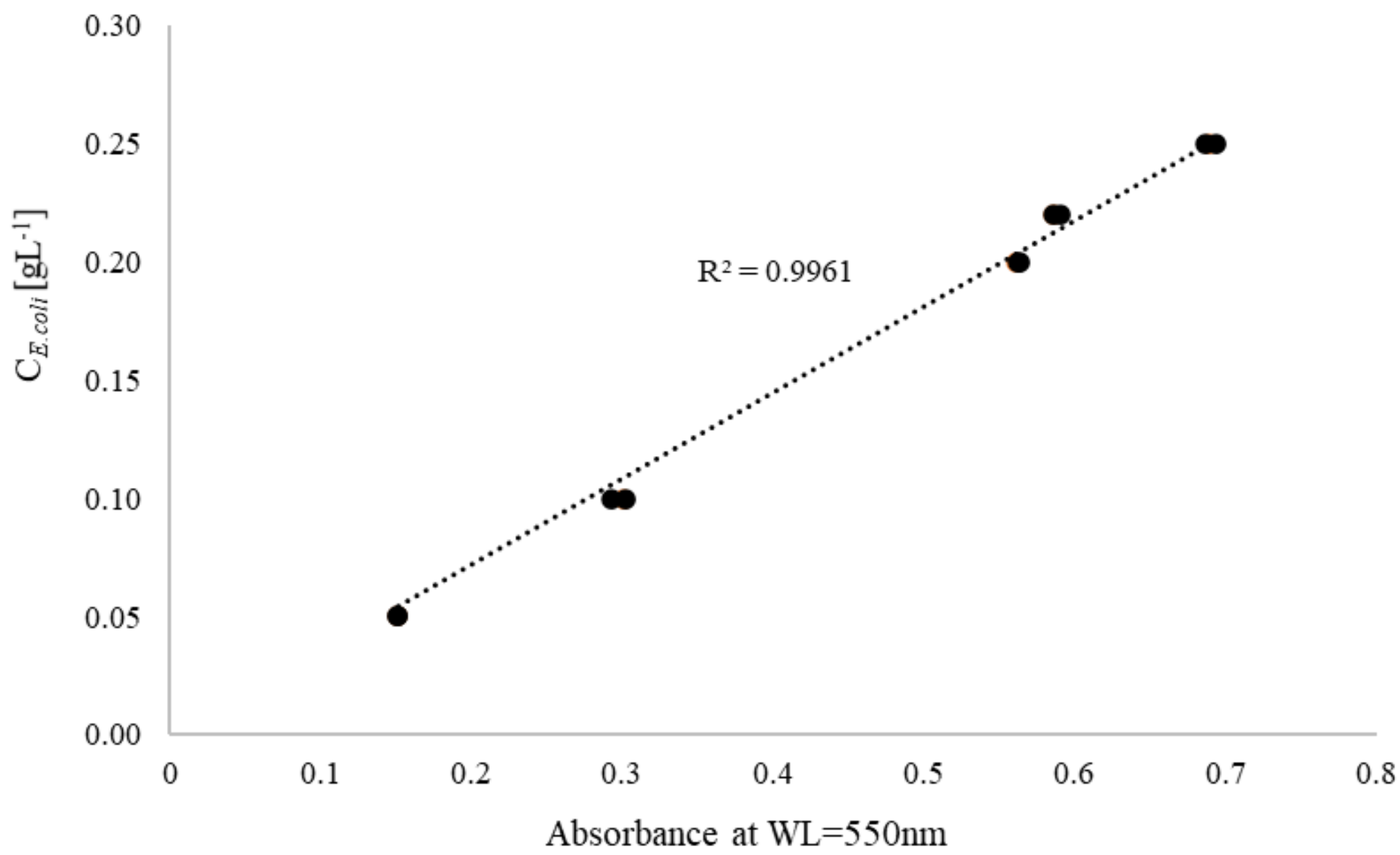

Figure S1. Standard curve for cell dry biomass

The standard curve was prepared by the cell suspension method. The cells suspension in basic M9 medium obtained after 24 hours of culturing was centrifuged (Hettich Unversal 320R, 20 min., 9000 rpm), the supernatant was discharged and the cells (precipitate) was washed by physiological salt

solution (0.9% NaCl) and again centrifuged. After decantation, the cells were placed in the laboratory dryer (Memmert, 45°C) for 24h. Different amounts of dry cells were suspended in distilled water, and the absorbance at 550 nm wavelength was measured. Each sample was prepared in three repetitions.

1.6. Glucose analysis

Glucose concentration was determined with the commercial colorimetric enzymatic test (Biomaxima, Poland). The intensity of the color was measured spectrophotometrically at 500 nm. The standard curve was given by the equation:

$$C_{glucose}=0.602A_{500} \quad \text{(S2)}$$

where: $C_{glucose}$ - is a concentration of glucose [gL^{-1}]; A_{500} - is an absorbance at 500 nm [-]. The standard curve is shown in Figure S2.

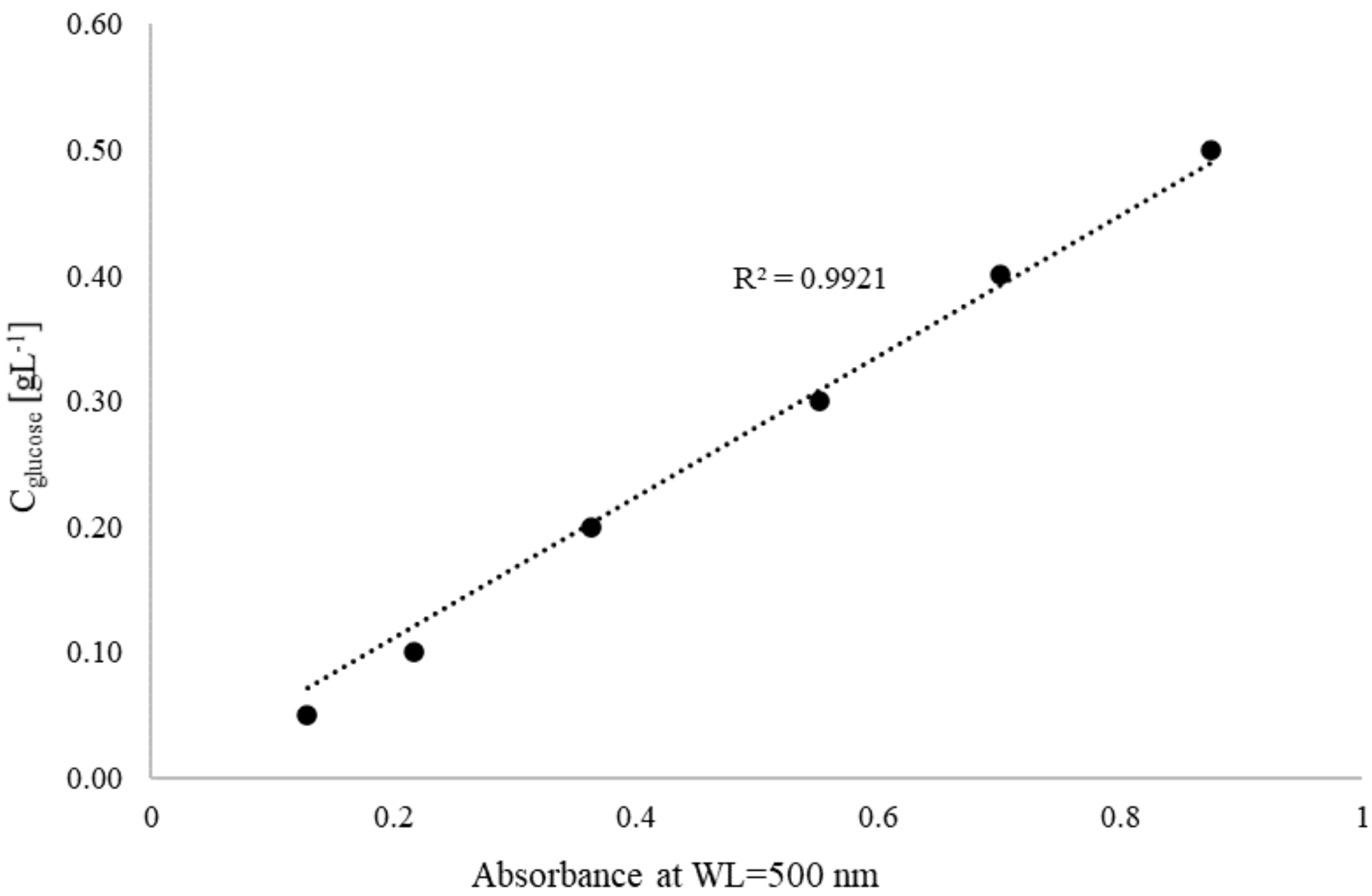

Figure S2. Standard curve for glucose concentration

The cell suspension samples (see *1.4. Culturing and sampling*) were centrifuged (3min, 6000 RPM). 50 μL of supernatant was added to 1 mL of analytical enzymatic reagent (Biomaxima, Poland) and incubated for 5 min at 37 °C. After this time the absorbance at 500 nm was measured.

Table S3. The Biomaxima reagent composition

Reagent	Concentration
Phosphate buffer pH=7.5	150 mmolL^{-1}
Glucose oxidase (GOD)	>20 kUL^{-1}
Peroxidase (POD)	>1.5 kUL^{-1}
4-aminoantipyrine (4-AA)	0.4 mmolL^{-1}
Phenol	5 mmolL^{-1}
Sodium azide	2 mmolL^{-1}
Non-reactive surfactants and stabilizers	13.9 mmolL^{-1}

The method based on the enzymatic reaction of glucose present in the sample with the enzymes in the Biomaxima reagent (the reagent composition is given in Table S3):

$$D - glucose + H_2O + O_2 \xrightarrow{GOD} gluconic\ acid + H_2O_2$$

$$H_2O_2 + 4 - AA + phenol \xrightarrow{POD} quinonemine + H_2O$$

The hydrogen peroxide combines with phenol and 4-aminoantypiryne to form a pink-colour complex. The color intensity measured photometrically is proportional to glucose concentration. The wavelength used to determine the concentration of glucose was selected on the spectrum of absorbance values of a solution containing 0.4 gL^{-1} of glucose, depending on the applied wavelength. The highest absorbance value was obtained for the wavelength of 500 nm (Figure S3).

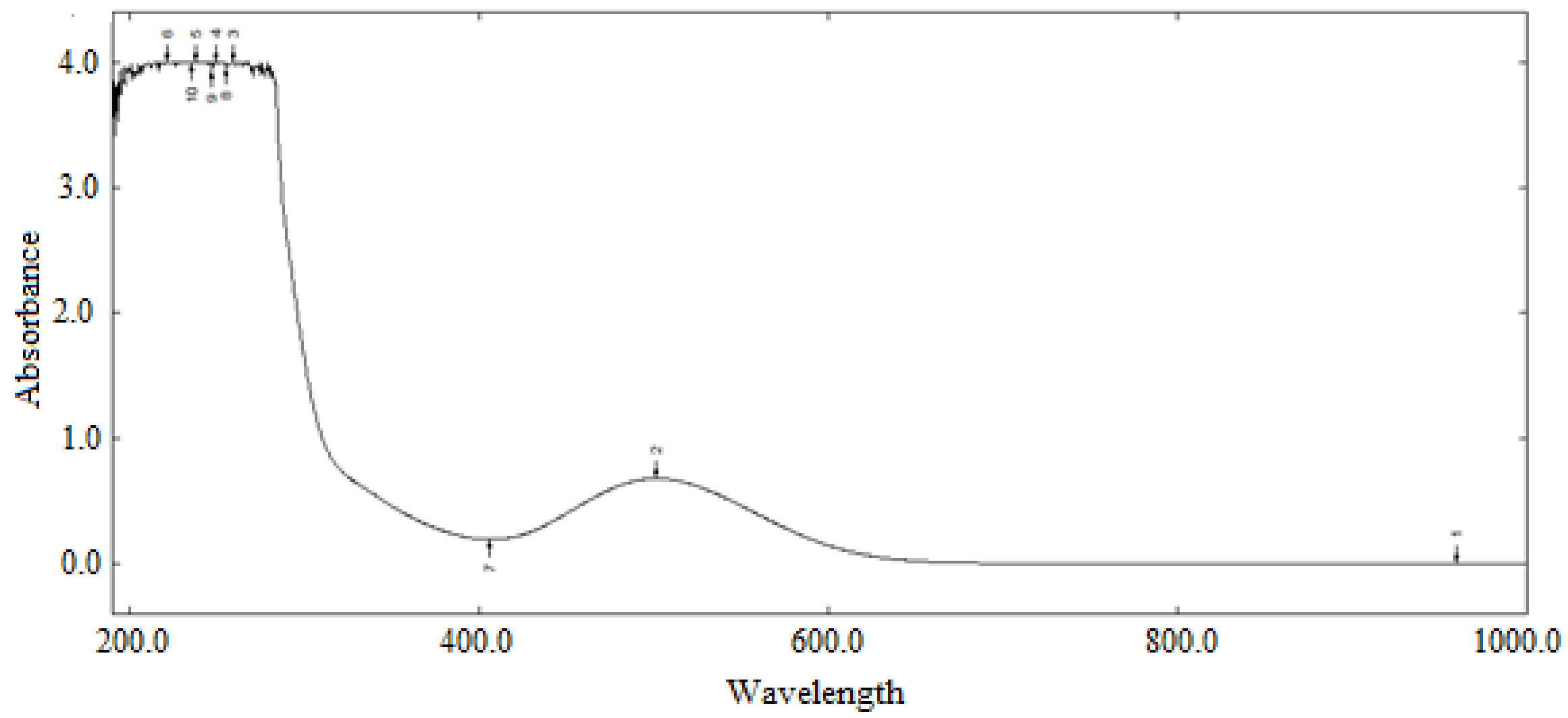

Figure S3. The spectrum of the absorbance value of the solution containing 0.4 g L^{-1} glucose depending on the wavelength used

1.7. Ammonium analysis

Ammonium concentration was determined with the cuvette test (Spectroquant, Merck, Germany). The intensity of the color was measured spectrophotometrically at 707 nm. The standard curve was given by the equation:

$$C_{NH4Cl}\ [gL^{-1}]=0.2030A_{707} \tag{S3}$$

where: C_{NH4Cl} - is a concentration of ammonia chloride [gL^{-1}]; A - is an absorbance at 707nm [-]. The background A_{707} was measured, and equal to 0.057, and subtracted from further measurements. The standard curve is shown in Figure S4.

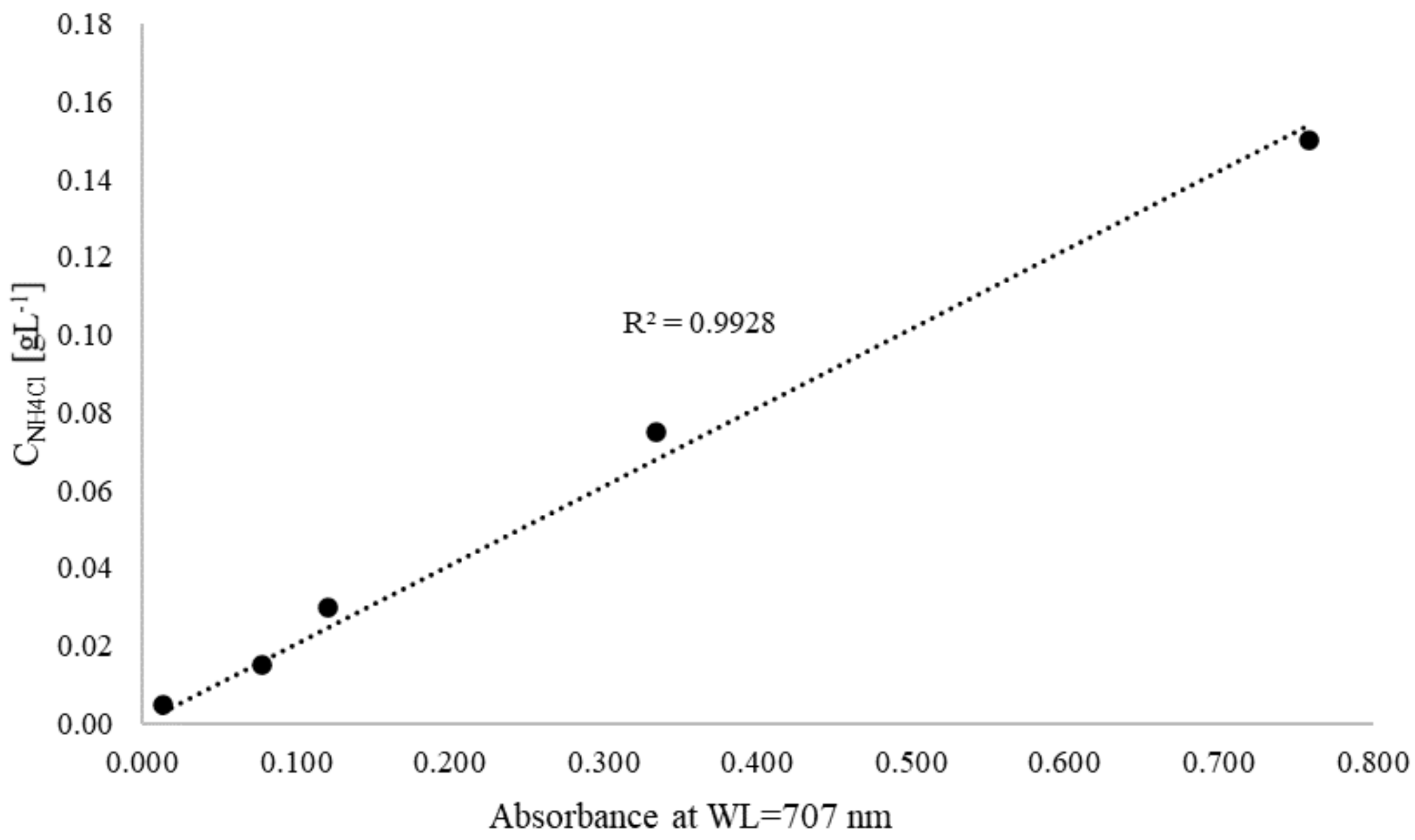

Figure S4. Standard curve for NH_4Cl concentration

The cell suspension samples (see *1.4. Culturing and sampling*) were centrifuged (3min, 6000 RPM). 100 μL of supernatant was added to 5 mL of analytical 1st reagent (NH_4-1, Spectroquant, Merck, Germany). To the mixture 1 level blue microspoon (in the cap of the NH_4-2 bootle – 2nd reagent) was added, mixed, and incubated for 10 min at room temperature. After this time the absorbance at 707 nm was measured.

This method determines a concentration of nitrogen in the form of ammonium nitrogen (NH_4-N), ammonium ($NH4^+$), ammonia nitrogen (NH_3-N), and ammonia (NH_3). In a strong alkaline solution ammonium nitrogen is present almost entirely as ammonia, which reacts with hypochlorite ions to form monochloramine. This in turn reacts with a substituted phenol to form a blue indophenol derivative that is determined photometrically. Due to the intrinsic yellow coloration of the reagent blank, the measurement solution is yellow-green to green color.

The wavelength used to determine the concentration of NH_4Cl was selected on the spectrum of absorbance values of a solution containing 0.4 gL^{-1} of NH_4Cl, depending on the applied wavelength. The highest absorbance value was obtained for the wavelength of 707 nm (Figure S5).

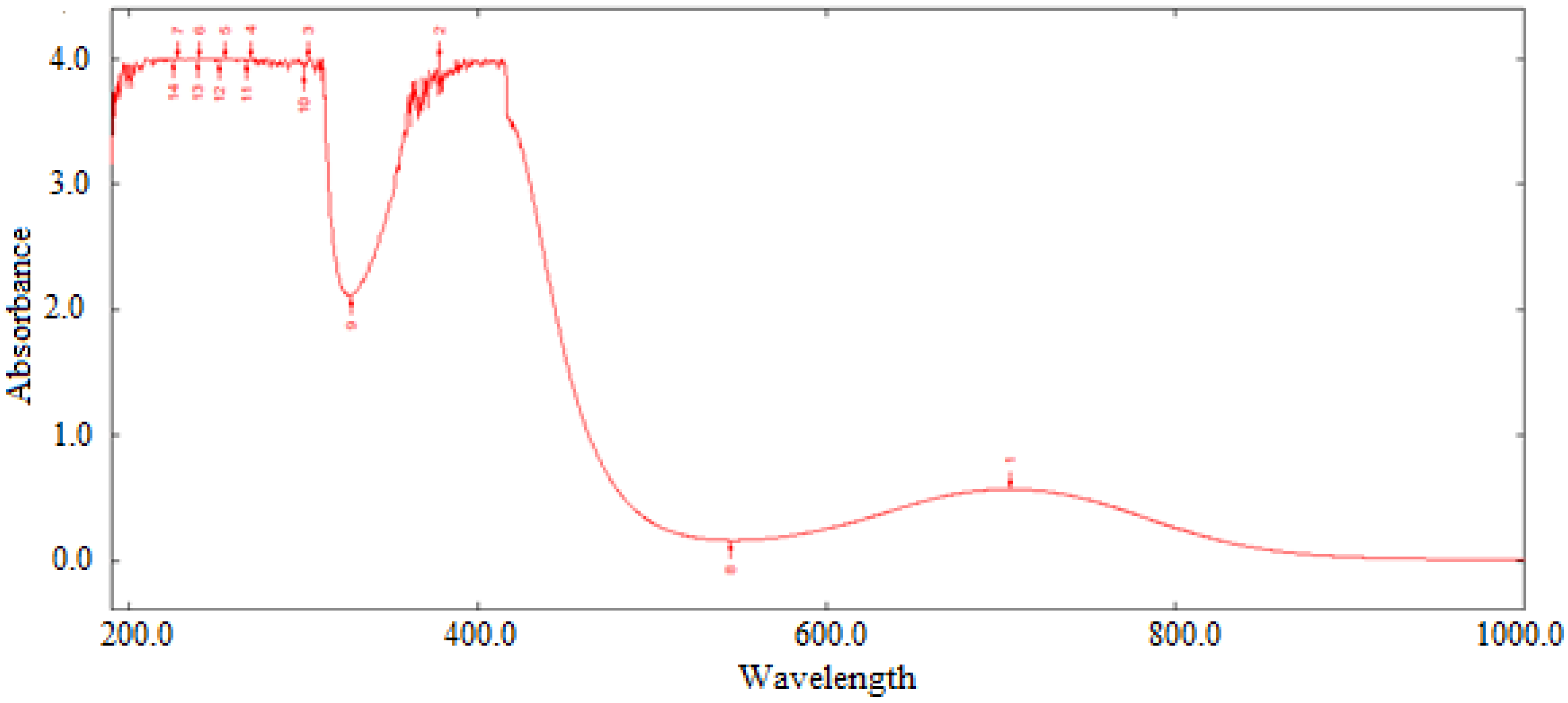

Figure S5. The spectrum of the absorbance value of the solution containing 0.1 g L^{-1} NH_4Cl depending on the wavelength used

2. Biomass composition

The content of C, H, N, and S in dry bacterial pellets obtained from C-limited and N-limited cultures was determined by CHNS Elemental Analyzer Vario EL Cube, with acetanilide as a standard (in the external laboratory). The results are given in Table S4.

Table S4. The mass content of C, H, N, and S in samples of bacterial pellet obtained in C-limited and N-limited growth conditions.

	Sample mass [mg]	C [%]	H[%]	N [%]	S[%]
C - limited	4.27	38.92	6.65	11.23	0.40
	4.44	38.88	6.70	11.21	0.42
N – limited	8.97	41.70	6.48	10.43	0.417
	3.97	40.66	7.16	10.61	0.42
	6.16	41.42	6.67	10.72	0.42

The content of each element in the sample can be recalculated for given molecular weights and expressed as a mean number of moles per one mole of carbon (C-mole). Therefore the C-mole of C-limited biomass is:

$$CH_{2.04}N_{0.25}S_{0.0040}R_C$$

and a C-mol of N-limited biomass is:

$$CH_{1.96}N_{0.22}S_{0.0038}R_N$$

where: R denotes the residual with unknown molecular weight. Probably the main component of both residuals is oxygen. Slightly lower H and N stoichiometric coefficients of *E. coli* biomass were determined previously for ammonium-limited and glucose-limited growth in a continuous reactor (Folsom and Carlson, 2015).

The molecular weight of C-limited and N-limited biomasses can be easily calculated as a ratio between sample mass and molar content of C in the sample. Therefore the mean molar weights of C-limited, and N-limited biomasses are equal $M_{WB\text{Clim}}$= 30.88 [g mol^{-1}] and $M_{WB\text{Nlim}}$= 29.11 [g mol^{-1}] respectively.

According to the DEB theory it can be assumed that biomass is built only from three components structure, C-reserve and N-reserve. Therefore, the biomass composition can be expressed as:

$$aCH_xO_yN_z + bCH_2O + cNH_3 = CH_kO_mN_n \qquad \text{(S4)}$$

where $aCH_xO_yN_z$ denotes a C-moles of structures with unknown stoichiometric coefficients x, y and z; bCH_2O - b C-moles of C-reserve which has the same elemental composition as glucose; cNH_3 - c

moles of N-reserve with the composition the same as ammonia; $CH_kO_mN_n$ – one C-mole of biomass in certain conditions with measured k and n values. Note that equation (S4) does not describe a chemical reaction, it is used to split biomass into three different components.

It can be assumed that the content of C-reserve in C-limited bacterial culture is equal to zero (b = 0), as well as N-reserve content in N-limited cultures (c = 0). Therefore, the composition of C-limited and N-limited biomass can be expressed as:

$$a_1CH_xO_yN_z + cNH_3 = CH_{2.04}N_{0.25}R_C \tag{S5}$$

$$a_2CH_xO_yN_z + bCH_2O = CH_{1.96}N_{0.22}R_N \tag{S6}$$

Note that the sulfur content was negligibly small and therefore it can be omitted, and the oxygen content hidden in residuals does not have to be known in further considerations. The mass balance for C, H, and N for C-limited biomass is given by the set of equations:

C: $a_1 = 1$ (S7)

H: $a_1x + 3c = 2.04$ (S8)

N: $a_1z + c = 0.25$ (S9)

and for N-limited biomass:

C: $a_2 + b = 1$ (S10)

H: $a_2x + 2b = 1.96$ (S11)

N: $a_zz = 0.22$ (S12)

The composition of structures is assumed to be constant. The set of six equations (S7) – (S12) can be simplified given that a_1= 1 to five equations with five unknowns a_2, b, c, x, and z. This set of nonlinear equations was solved using the Trust-Region-Dogleg algorithm in Matlab. The results were: a_2 = 0.99, b = 0.0149, c = 0.027, x = 1.96, and z = 0.22. Therefore the composition C-mol of structures can be expressed as $CH_{1.96}O_yN_{0.22}$. The stoichiometric coefficient y was assumed to be equal to 0.45 based on the (Folsom and Carlson, 2015) and used only in material balance calculations. The molecular weight of C-mol of structure $M_{W\mathrm{Mv}}$ can be calculated from the equations:

$$M_{W\mathrm{Mv}} = \frac{M_{W\mathrm{BClim}} - cM_{WEN}}{a_1} \tag{S13}$$

and/or

$$M_{W\mathrm{Mv}} = \frac{M_{W\mathrm{BNlim}} - bM_{WEC}}{a_2} \tag{S14}$$

where M_{WEC}, and M_{WEN} are molecular weight of C-moles of C-, and N-reserves equal to the molecular weight of C-mol of glucose and ammonium ion, 30.0267 and 18 [g mol^{-1}] respectively.

The mean value of molecular weight of C-mol of structure $M_{W\mathrm{Mv}}$was equal to 29.76 [g mol^{-1}], and was used along with M_{WEC} and M_{WEN} for mass-moles recalculation within the DEB model. The mean biomass C-mol molecular weight was equal to M_{WB} = 30.00 [g mol^{-1}] and was used within Monod's model.

It should be noted that the solution of six equations (S7) – (S12) is highly sensitive to the stoichiometric coefficients of C-mol of biomass (S5) and (S6). Therefore the results can be fraught with considerable error.

3. Shock limitation and reserves kinetics

The shock limitation experiment was designed to investigate the reserve density kinetics and to verify the growth and maintenance constraints. The growing cells were transferred to the medium where one of substrates, glucose or ammonia, was missing and therefore could not be used to supply reserves. The model predictions were compared with measured values. The simulation shows the reserves, structure, and biomass dynamics before, and right after the growing cells were transferred to the medium without C-substrate (raw A) or N-substrate (raw B) (Fig. S6).

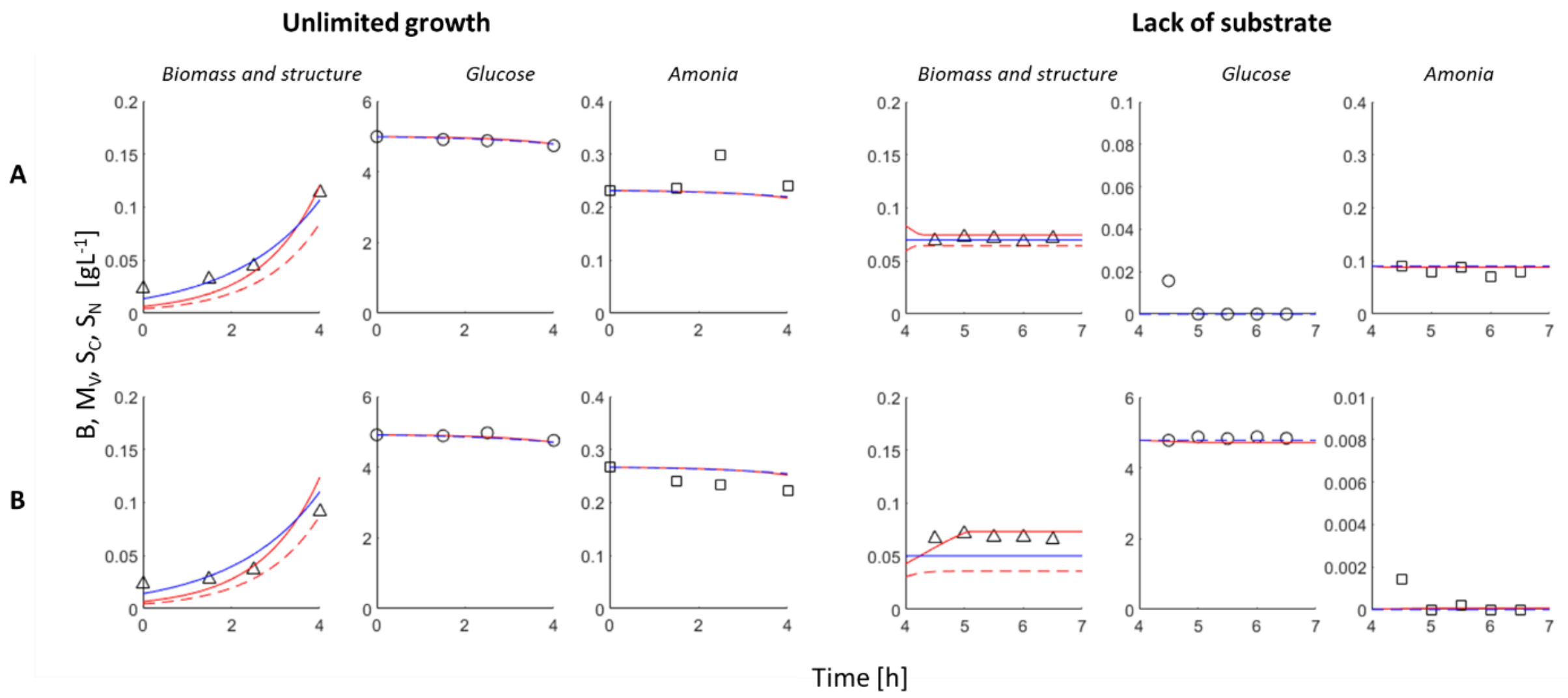

Figure S6. DEB and Monod's model simulation to experimental data obtained in shock limitation experiments. The simulation shows the reserves, structure, and biomass dynamics before and after the growing cells were transferred to the medium without C-substrate (raw A) or N-substrate (raw B). Model parameter values listed in Tab. 1 were used.

In case when the bacteria cells were transferred to the medium without glucose, the model predicts a decrease in biomass due to the decrease in C-reserve density and gentle increase in structures (Fig. S6). In the case when bacteria were transferred into a medium without ammonia small increase in biomass and C-reserves was predicted. The growth and maintenance can continue until the N-reserve is sufficiently filled. N-reserve density is low and therefore it has a low influence on total biomass. Nevertheless, the model prediction and constraints used were confirmed only by the data in time intervals where the biomass does not change anymore (Fig. S6).

4. Mass balance

The material balance for each of the four main elements C, H, O, and N is given by flowing equation (S15):

$$0 = \mathbf{n_O}\mathbf{J_O} - \mathbf{n_M}\mathbf{J_M} \tag{S15}$$

where $\mathbf{J_O}$ is a vector of total 'organic' fluxes $\dot{J}_*$ (capital letter J, * stands for substrates S_C, S_N, reserves, E_C, E_N or structure V) [mol h^{-1}]. The name 'organic' is used in the DEB book (Kooijman, 2010), however, the set of fluxes in vector $\mathbf{J_O}$ describes general changes of variables used in the model: substates, reserves, and structures (no matter if they are actually organic or not, like ammonia).

$$\mathbf{J_O} = \begin{bmatrix} \dot{J}_{S_C} \\ \dot{J}_{S_N} \\ \dot{J}_{E_C} \\ \dot{J}_{E_N} \\ \dot{J}_V \end{bmatrix}_{5\times1} = \begin{bmatrix} \mathrm{V}\frac{d}{dt}S_C \\ \mathrm{V}\frac{d}{dt}S_N \\ \frac{d}{dt}M_{E_C} \\ \frac{d}{dt}M_{E_N} \\ \frac{d}{dt}M_V \end{bmatrix}_{5\times1} \tag{S16}$$

Note that reserves are expressed in absolute values M_{E_i} [mol h^{-1}], not as reserves densities, therefore:

$$\frac{d}{dt}M_{E_i} = \left(j_{E_iA} - j_{E_iC} + \kappa_{E_i} j_{E_iR}\right)M_V \tag{S17}$$

Vector $\mathbf{J_O}$ can be expressed using specific fluxes j_* [mol C-mol$_{MV}^{-1}$ h^{-1}] as follows:

$$\mathbf{J_O} = \begin{bmatrix} -y_{SE_C} & 0 & 0 & 0 & y_{SE_C}\kappa_{S_C}\left(1-\kappa_{E_C}\right) & 0 & -y_{SE_C}\kappa_{S_C}\left(1-\kappa_{E_C}\right)y_{E_CV} \\ 0 & -y_{SE_N} & 0 & 0 & 0 & y_{SE_N}\kappa_{S_N}\left(1-\kappa_{E_N}\right) & -y_{SE_N}\kappa_{S_N}\left(1-\kappa_{E_N}\right)y_{E_NV} \\ 1 & 0 & -1 & 0 & \kappa_{E_C} & 0 & -\kappa_{E_C}y_{E_CV} \\ 0 & 1 & 0 & -1 & 0 & \kappa_{E_N} & -\kappa_{E_N}y_{E_NV} \\ 0 & 0 & 0 & 0 & 0 & 0 & 1 \end{bmatrix}_{5\times7} \times \begin{bmatrix} \dot{j}_{E_CA} \\ \dot{j}_{E_NA} \\ \dot{j}_{E_CC} \\ \dot{j}_{E_NC} \\ \dot{j}_{E_CG} \\ \dot{j}_{E_NG} \\ \dot{j}_G \end{bmatrix}_{7\times1} \times M_V \tag{S18}$$

which directly connects this part with the main model equations (10 – 12). $\mathbf{n_O}$ is the stoichiometric coefficients matrix for 'organic' substances:

$$\mathbf{n_O} = \begin{bmatrix} 1 & 0 & 1 & 0 & 1 \\ 2 & 3 & 2 & 3 & 1.96 \\ 1 & 0 & 1 & 0 & 0.45 \\ 0 & 1 & 0 & 1 & 0.22 \end{bmatrix}_{4\times5} \tag{S19}$$

$\mathbf{J_M}$ is a vector of minerals (or metabolites) fluxes [mol h^{-1}]:

$$\mathbf{J_M} = \begin{bmatrix} j_{CO_2} \\ j_{H_2O} \\ j_{O_2} \\ j_{NH_3} \end{bmatrix}_{4\times1} \qquad \text{(S20)}$$

$\mathbf{n_M}$ is the stoichiometric coefficients matrix for minerals (metabolites):

$$\mathbf{n_M} = \begin{bmatrix} 1 & 0 & 0 & 0 \\ 0 & 2 & 0 & 3 \\ 2 & 1 & 2 & 0 \\ 0 & 0 & 0 & 1 \end{bmatrix}_{4\times4} \qquad \text{(S21)}$$

The equation (S15) can be solved to calculate the total mineral fluxes:

$$\mathbf{J_M} = -\mathbf{n_M}^{-1}\mathbf{n_O}\mathbf{J_O} \qquad \text{(S22)}$$

The equation (S22) was solved numerically with model equations (10 – 12) for estimated parameter values giving the total produced amounts of metabolites (per liter of growth medium) in time. An example of metabolites production/consumption analysis was prepared for the C-limitation scenario with an initial glucose concentration of 1 gL-1 and is presented in Figure S7.

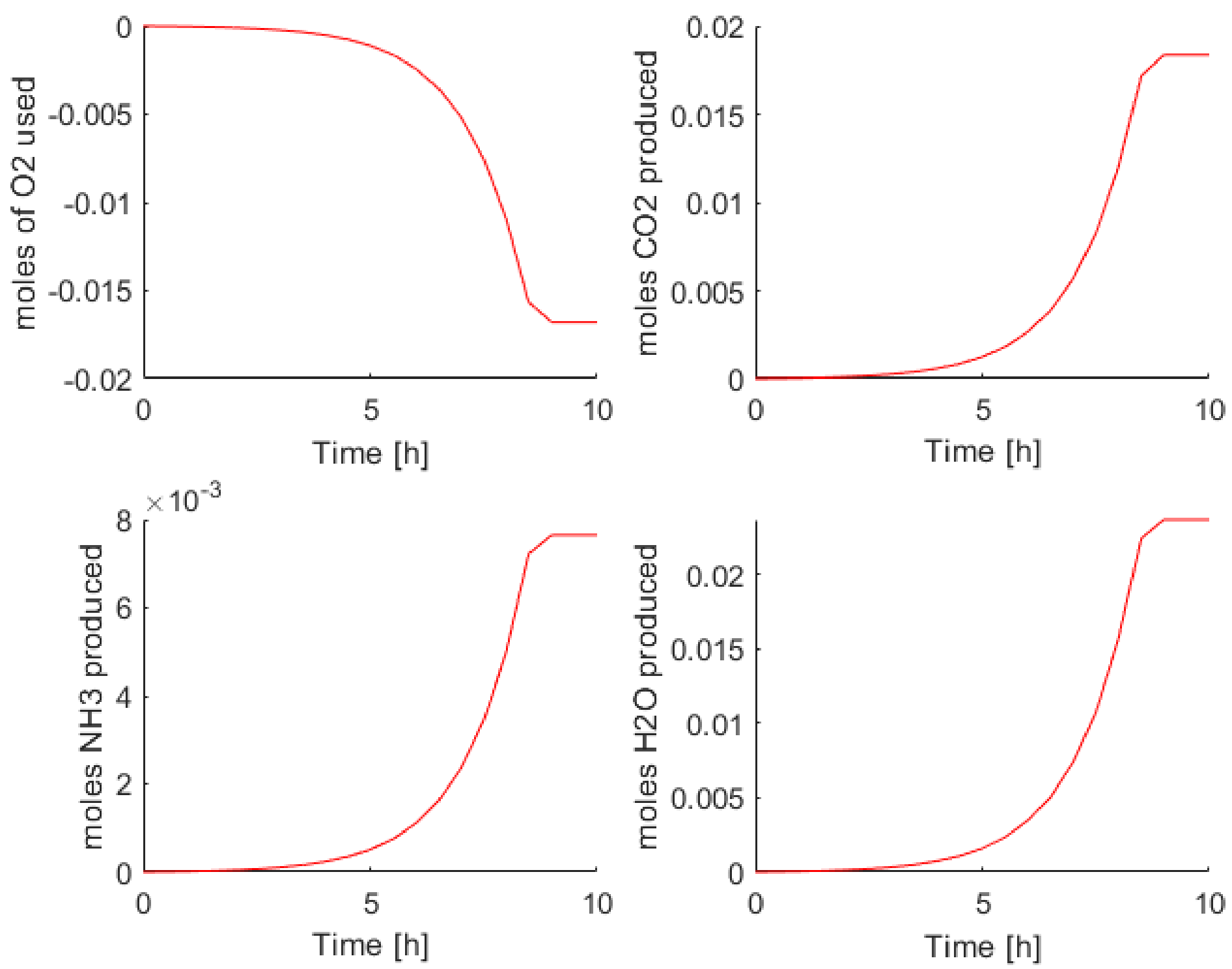

Figure S7. Amounts of metabolites produced in time.

Moreover, the mass balance was checked using the equation:

$$\mathbf{n_O}\mathbf{J_O} + \mathbf{n_M}\mathbf{J_M} = initial\ value\ of\ element\ C, H, O, N \qquad \text{(S23)}$$

The total amounts of elements during the process for considered example are shown in Figure S8. Their values do not change indicating the mass balance is preserved.

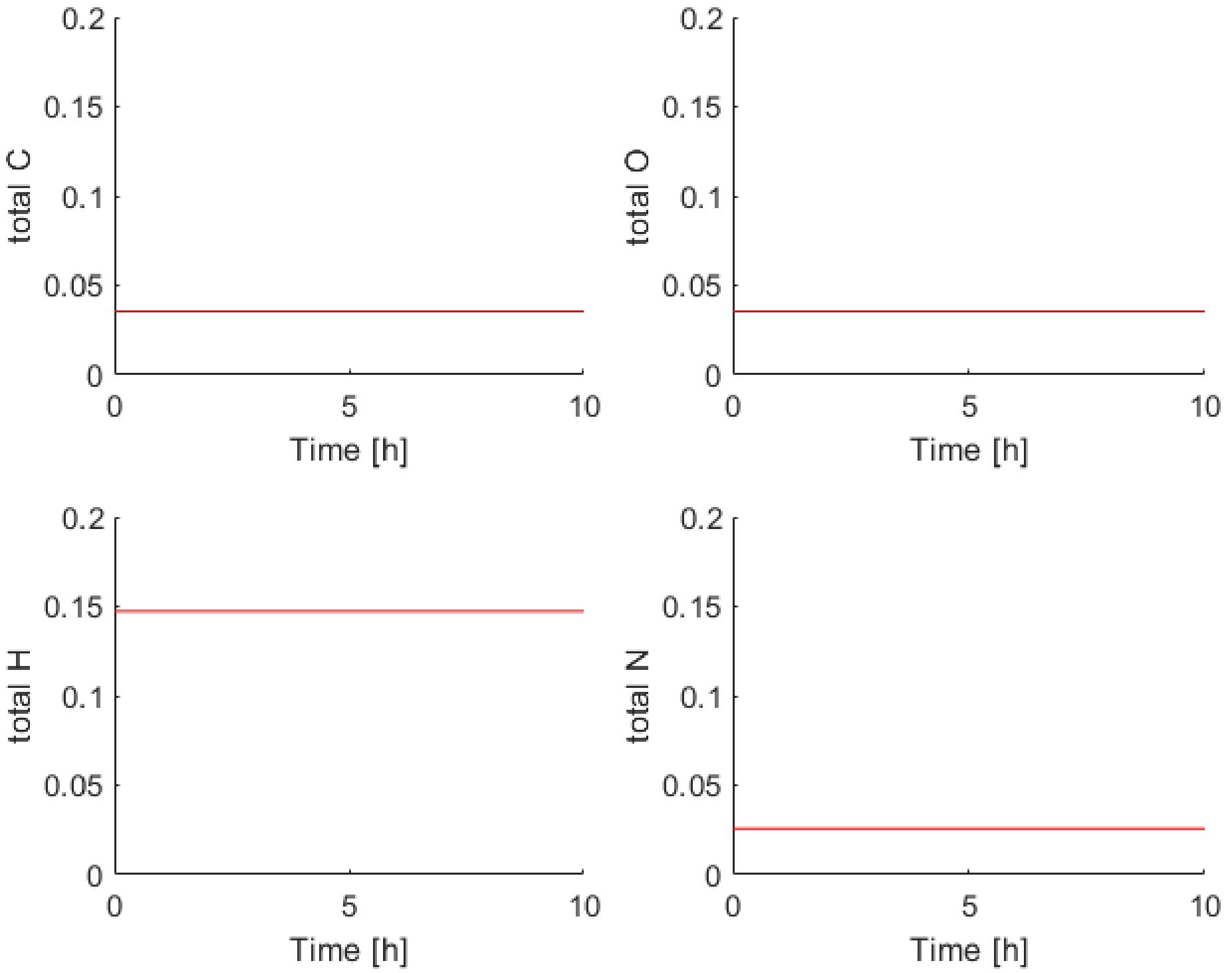

Figure S8. Amounts of metabolites produced in time.

5. Parameters estimation

5.1. Units conversion

The DEB model and Monod's model were fitted to the data, which include biomass, glucose and ammonia concentrations expressed in [gL^{-1}]. Hoverer both model variables were expressed in [mol L^{-1}]. Therefore in each step of loss function minimization, the numerical solutions of differential equations – the model variables were converted from [mol L^{-1}] to [gL^{-1}] using molecular weights as conversion factors. The molecular weights of glucose and ammonia were $M_{W\mathrm{SC}}$= 30.0267 [g mol^{-1}] and $M_{W\mathrm{SN}}$= 18 [g mol^{-1}] respectively and were used in both models. The molecular weight of C-mol of structure in DEB model $M_{W\mathrm{Mv}}$was equal to 29.76 [g mol^{-1}], and the molecular weight of C-reserve and N-reserve, M_{WEC} and M_{WEN} respectively, were equal to those of glucose and ammonia. C-reserve and N-reserve densities were recalculated from [mol mol^{-1}] to [g g^{-1}] using the ratios $M_{W\mathrm{SC}}/M_{W\mathrm{Mv}}$ and $M_{W\mathrm{SN}}/M_{W\mathrm{Mv}}$. The molecular weight of biomass in Monod's model was equal to M_{WB} = 30.00 [g mol^{-1}].

Note that it was necessary to convert [mol L^{-1}] to [gL^{-1}] because the total biomass concentration in the DEB model is a sum of structure mass and C and N reserves masses per unit volume. Note that the masses of different substances can be added, however moles cannot.

5.2. Estimation algorithm

The loss function was minimized using the Nelder-Mead Simplex Method in Matlab (Lagarias et al., 1998). The set of DEB model and Monod's model differential equations were solved using the Runge-Kutta method in each step of loss function minimization. In each step of the Runge-Kutta method, the value $\dot{r}$ in the DEB model had to be found by solving the equation:

$$0 = \left[\sum_{i\in(C,N)}\left(\frac{m_{E_i}(k_E-\dot{r})-j_{E_iM}}{y_{E_iV}}\right)^{-1} - \left(\sum_{i\in(C,N)}\frac{m_{E_i}(k_E-\dot{r})-j_{E_iM}}{y_{E_iV}}\right)^{-1}\right]^{-1} - \dot{r} \qquad \text{(S24)}$$

Trust-Region-Dogleg algorithm was used to solve it. All calculations were performed in Matlab.

5.3. Estimation procedure

The DEB model parameters j_{E_CAm}, j_{E_CM}, j_{E_NAm}, j_{E_NM}, and $\dot{k}_E$ are difficult to be identified simultaneously from given datasets. Therefore the procedure estimation was divided into two steps.

First, it was assumed that only C-substrate can influence the growth rate, therefore the N-substrate and N-reserves dynamics were omitted. In that case, the DEB model can be highly simplified. The specific growth rate is given by the equation:

$$\dot{r} = \frac{m_{E_i}(\dot{k}_E - \dot{r}) - j_{E_iM}}{y_{E_iV}} \tag{S25}$$

which do not have to be solved numerically like equation (S15). The parameters j_{E_CAm}, j_{E_CM}, and $\dot{k}_E$ were estimated using the nonlinear least-squares method with stating values for these parameters 2.2, 0.6, and 4.9 respectively. The results were 1.893, 0.6719, 5.0654. Analogically for N-substrate the values of j_{E_NAm}, j_{E_NM}, and $\dot{k}_E$ were estimated. Starting values were 0.28, 0.00075, 5, and the results were 0.1894, 0.0009, 5.4961. Obtained values of j_{E_iAm}, and j_{E_iM}, and were used as starting values in further estimation procedures. The reserve turnover rate $\dot{k}_E$ = 5.3 was determined as a mean of the two mentioned estimates and used further as a constant.

Second, the values of four parameters j_{E_iAm},and j_{E_iM} were estimated simultaneously from the whole set of data obtained in the main experiment.

The Monod's model (equations (13) – (16)) parameters μ_{max}, Y_{XS_C}, and Y_{XS_N} were fitted simultaneously to given datasets: Starting values of these parameters were 0.4, 0.5, and 6.0.

5.4. Goodness of fit

The goodness of fit was described by the adjusted coefficient of determination ($R^2{}_{adj}$), and root mean square error (RMSE). The adjusted coefficient of determination was given by the equation (S26):

$$R^2{}_{adj} = 1 - \frac{n-1}{n-p}\frac{SSE}{STOT} \tag{S26}$$

where - n – is the number of data points, p – is the number of estimated parameters, SSE – is a sum of squared errors between the model and data points (note that here it is not a weighted sum of squared errors), $STOT$ - is a sum of squared errors between the mean value of the datapoints and the datapoints. The root-mean- squared error was given by the equation (S27):

$$RMSE = \sqrt{\frac{SSE}{df}} \tag{S27}$$

where: df – denotes the number of degrees of freedom equal to $n - p$.
Moreover, nonsimultaneous prediction intervals for the next observation were plotted along with model's solution.

6. Synthesizing unit

In this study, we assume that glucose and ammonia are stored in different reserves. These reserves are mobilized and the occurring fluxes are combined to synthesize new structure. The process of structure synthesis from two substrates can be described as a parallel and complementary process. The scheme of this kind of process for two substrates A and B and one product C is presented in Figure S9.

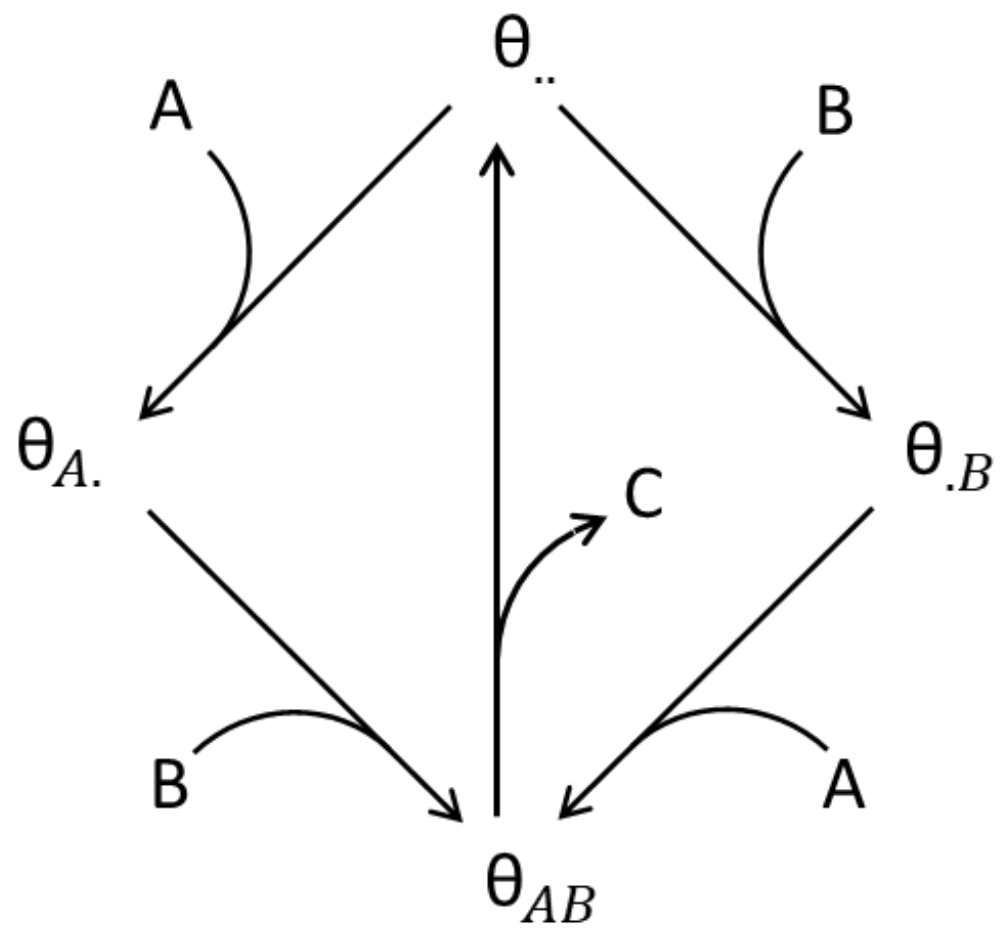

Figure S9. The scheme of parallel and complementary synthesis.

The symbol θ denotes an enzyme that is not changed during this reaction. The synthesis of C occurs only when both substrates are bonded to the enzyme. It should be noted that the main idea of this kind of process is that the order in which A and B are bonded does not matter.

The different types of interactions of substrates A and B in transformation into product C can be found in (Kooijman, 2010).

References

Folsom, J.P., Carlson, R.P., 2015. Physiological, biomass elemental composition and proteomic analyses of *Escherichia coli* ammonium-limited chemostat growth, and comparison with iron- and glucose-limited chemostat growth. Microbiology (N Y) 161, 1659–1670. https://doi.org/10.1099/mic.0.000118

Kooijman, S.A.L.M., 2010. Dynamic Energy Budget theory for metabolic organisation, Third Edit. ed. Cambridge University Press, New York. https://doi.org/10.1017/CBO9780511805400

Lagarias, J.C., Reeds, J.A., Wright, M.H., Wright, P.E., 1998. Convergence Properties of the Nelder-Mead Simplex Method in Low Dimensions. SIAM Journal on Optimization 9, 112–147. https://doi.org/10.1137/S1052623496303470
